# Cities can benefit from complex supply chains

**DOI:** 10.1038/s42949-023-00100-5

**Published:** 2023-03-29

**Authors:** Nazlı B. Doğan, Alfonso Mejia, Michael Gomez

**Affiliations:** Department of Civil and Environmental Engineering, The Pennsylvania State University, University Park, PA USA

**Keywords:** Complex networks, Civil engineering, Economics, Sustainability

## Abstract

Supply chain complexity is perceived to exacerbate the supply disruptions or shocks experienced by a city. Here, we calculate two network measures of supply chain complexity based on the relative number—horizontal complexity—and relative strength—vertical complexity—of a city’s suppliers. Using a large dataset of more than 1 million annual supply flows to 69 major cities in the United States for 2012–2015, we show that a trade-off pattern between horizontal and vertical complexity tends to characterize the architecture of urban supply networks. This architecture shapes the resistance of cities to supply chain shocks. We find that a city experiences less intense shocks, on average, as supplier relative diversity (horizontal complexity) increases for more technologically sophisticated products, which may serve as a mechanism for buffering cities against supply chain shocks. These results could help cities anticipate and manage their supply chain risks.

## Introduction

In our highly interconnected world, cities fundamentally depend on supply chain networks to function and thrive^1–4^. Supply networks have become increasingly complex over time due, among other factors, to the intensification of global trade and changes in supply chain management practices^5,6^, such as just-in-time supplies and lean replenishments. These management strategies promote efficiency but may also undermine network redundancy and resiliency^7^. The complexity of supply chains is widely perceived to exacerbate supply disruptions or shocks^3,4^. In ecological theory, however, the stability of ecosystems is related to their complexity^8^, and empirical analyses of ecological^8,9^ and human^10,11^ networks show that complexity often accompanies diversity, which can serve as a stabilizing force^10,12^. By analogy, we contend that supply chain complexity linked to supplier diversity may serve as a mechanism for protecting cities against supply chain shocks.

Many internal characteristics of cities (e.g., number of employees and length of roads) have been shown to vary systematically with urban population in a universal power-law relationship^13^. These findings, however, do not explicitly consider the fact that cities coexist and interact with other cities and regions in a network system^14,15^. Thus, it is unclear whether urban supply networks share a common pattern across cities with different characteristics. Although previous studies have investigated the association between supply chain complexity and supply chain shocks at the company level^3,4^, this association is not well known for cities. Furthermore, a network’s complexity is determined by its topological and interaction strength patterns, which tend to shape the network’s resiliency^16,17^. Therefore, understanding how the network architecture of supply chains varies across cities, and how that architecture relates to a city’s ability to resist or buffer supply chain shocks, is crucial for predicting and managing supply chain risks.

Measuring supply chain complexity is generally complicated by a lack of visibility of upstream or higher-tier suppliers^1,5^. Typically, a company only has complete visibility of immediate or first-tier suppliers^18^. Here, we derive a network-based index of supply chain complexity that uses data about a city’s immediate suppliers to infer the complexity of its higher-tier suppliers. The index assumes that the level of technological sophistication or complexity of a product reflects its level of upstream supply chain complexity, which is a reasonable assumption because more complex products (e.g., electronics) tend to have more component parts and require more upstream supply chain stages for production than basic ones (e.g., coal)^19^. To rank the complexity of city import products in the United States, we use a dimensionality reduction algorithm that has been shown to be useful for this task^20–22^ as well as for predicting economic growth^23^ and identifying economic specialization patterns^20^ at the city level. Thus, our data-driven measure of supply chain complexity is based on both the structure of supply networks and the sophistication of products, which according to supply chain theory are key factors driving supply chain complexity^3,18,24^. In addition, our measure has the advantage that it can be calculated using supply chain data for first-tier suppliers.

## Results and discussion

### Network measures of supply chain complexity

Using a large dataset of more than 1 million annual supply flows from 2012 to 2015^25^, the years with available data, we determine the supply chain complexity of 115 regions covering the entire United States, including 69 major cities ranging in population from ~2 × 10^5^ to 2 × 10^7^ in the year 2012 (Supplementary Data 1). In 2012, these cities accounted for ~68% of the total population and ~74% of the total gross domestic product in the United States. The dataset also includes international supply flows from eight world regions (see Methods).

**Fig. 1:**
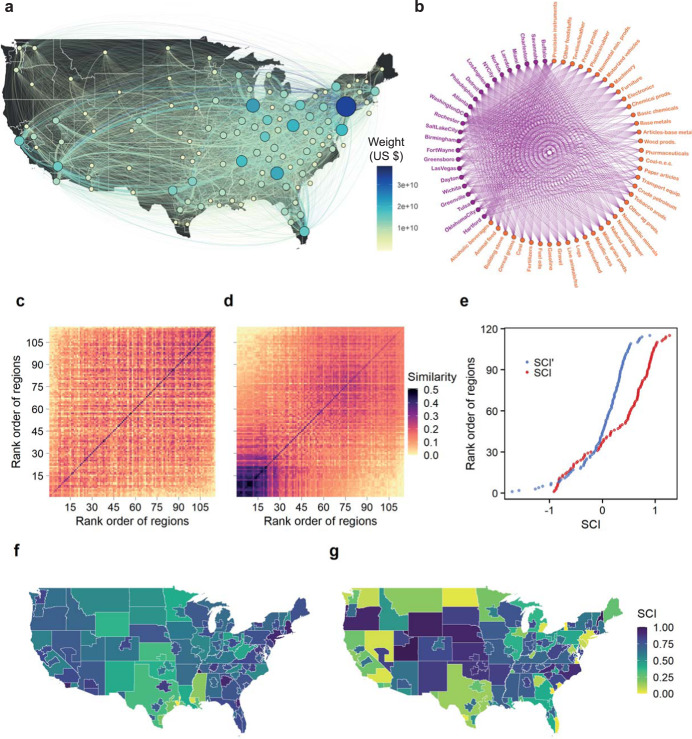
Workflow created to calculate supply chain complexity indices. **a** Illustration of the supply network for pharmaceutical products in the United States in 2012. Although not shown for clarity, the network includes international connections to the rest of the world via eight different world regions. The nodes in the network correspond to the 115 United States regions in our dataset. The node size represents the total supply inflows to a node (in-strength), and links represent the supply flows (in dollar units) between nodes, with the direction of flow being clockwise. **b** Binary bipartite network of product-region pairs (only the 25 most populous cities are shown) derived from all spatial supply networks in 2012. Similarity matrix for SCI (**c**) and SCI′ (**d**) derived from the product-region bipartite network. **e** Rank order of cities and regions with respect to their unstandardized SCI and SCI′ values. Mapping of normalized SCI (**f**) and SCI′ (**g**) values for cities and regions in the United States.

To implement the dimensionality reduction algorithm, we start with the supply network for each of the 39 product categories in our dataset (Fig. 1a illustrates one of these supply networks). These 39 product categories capture the complete product economy of the United States, although at a coarse level of aggregation. We combine the 39 supply networks into a single product-region, binary bipartite network (Fig. 1b and Supplementary Fig. 1), with links assigned using a location quotient (LQ) equation. For a specific product, LQ is used to calculate the concentration of supply inflows (connections) in a region as the share of that region’s supply inflows (connections) relative to the national share (Methods). The product–region pairs with LQ ≥ 1 are assigned a link in the bipartite network (Fig. 1b and Supplementary Figure 2). This condition highlights key industries that differentiate city supply chain structure^26^.

We distinguish between two key structural dimensions of supply chain complexity, termed vertical and horizontal complexity^4^. Vertical complexity is calculated using the shares of a city’s supply inflows whereas horizontal complexity is based on the shares of a city’s number of supplier connections (Methods). This results in two estimates of our supply chain complexity index (SCI); hereafter, SCI is used to indicate vertical complexity and SCI′ to indicate horizontal complexity. The calculation of SCI is similar to that of the economic complexity index^20^ with the main difference being that SCI is based on supply flows rather than production flows. By being based on the supplier connections, the calculation of SCI′ differs more fundamentally from that of the economic complexity index (Methods). Thus, SCI and SCI′ characterize the interaction strength and topological patterns, respectively, of urban supply chains. Furthermore, Spearman’s rank correlation is moderate (*R* = 0.53, *P* < 0.001) between the ranking of the products’ complexity used to derive the SCI and SCI′ (Methods), indicating that the indices tend to rank the same product categories as high or low, facilitating comparison between SCI and SCI′.

To visually interpret the algorithm^20^, we employ the similarity matrices (Fig. 1c, d), which are used to calculate the indices (Methods). The rows and columns of the similarity matrices are in ascending order according to the SCI (Fig. 1c) and SCI′ values (Fig. 1d). The algorithm places cities with similar supply network structures closer together and cities with dissimilar structures farther apart. At SCI or SCI′ = 0 (Fig. 1e), the algorithm aims to partition cities into two main similarity groups^20^, although here this partition is more evident for SCI′ (Fig. 1d) than for SCI (Fig. 1c). Despite the spatial heterogeneity of the indices (Fig. 1f, g), some distinctive features emerge; for example, SCI values tend to be high for large cities and SCI′ low.

### Relationships between supply chain complexity and local city and supply network characteristics

**Fig. 2:**
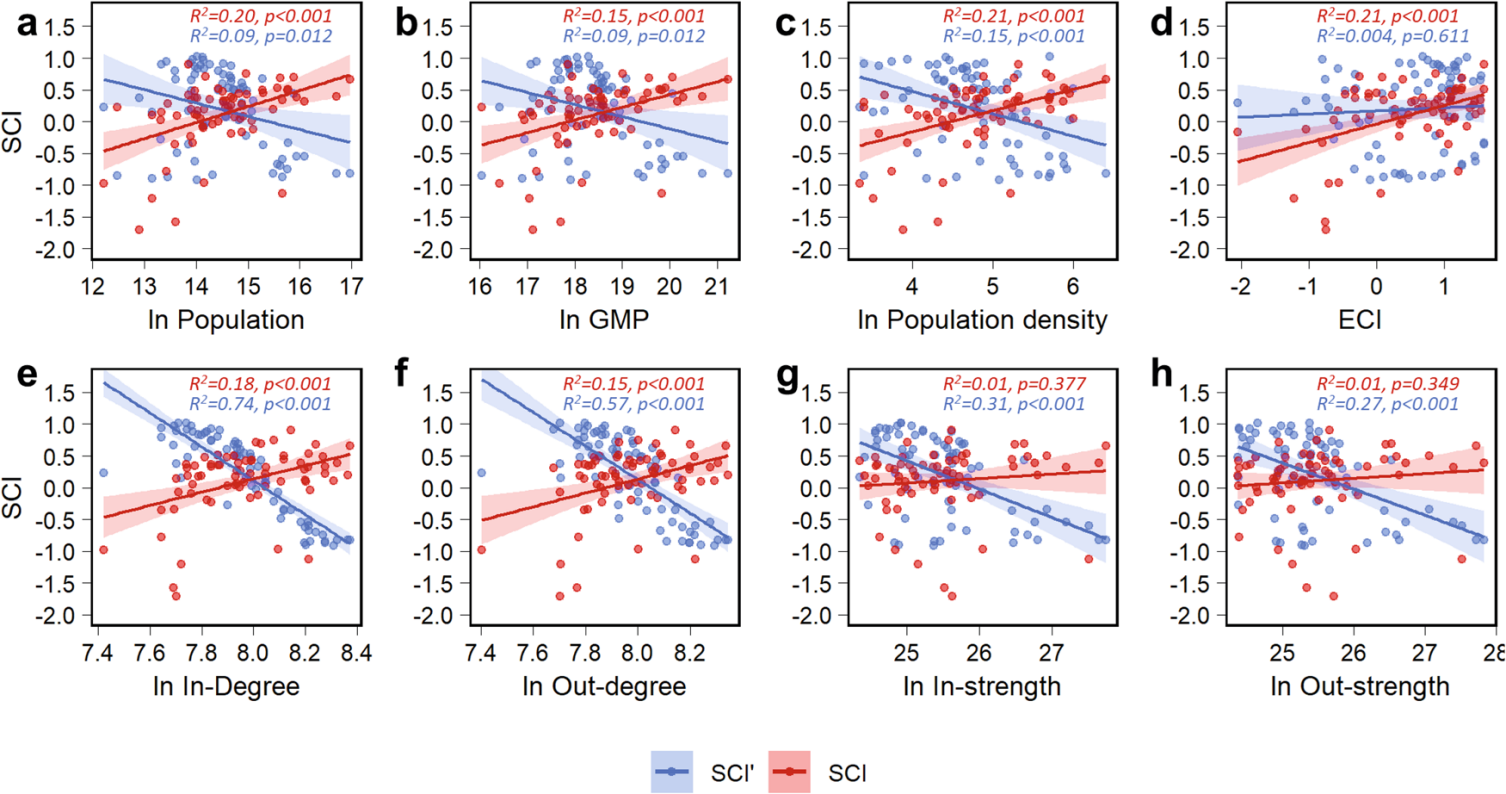
Trade-off pattern between horizontal and vertical supply chain complexity. **a**–**d** Relationships between supply chain complexity and local city characteristics for 2012 [ln population, ln gross metropolitan product (GMP), ln population density, and standardized economic complexity index (ECI)]. **e**–**h** Relationships between supply chain complexity and local supply network characteristics for 2012 [in-degree (total number of suppliers), out-degree (total number of exporting regions), in-strength (total inflows), and out-strength (total outflows); all in ln scale]. Local supply network characteristics are calculated by aggregating all the supply networks for 2012. For each plot, *n* = 69 with each point representing a city. The best linear fits are also shown, together with their 90% confidence intervals, *R*^*2*^ values, and *p* values.

We find that SCI values tend to increase as SCI′ values decline (Fig. 2). This trade-off pattern between SCI and SCI′ characterizes how the architecture of urban supply networks varies across cities. The pattern is observed across different local city (Fig. 2a–d) and local supply network (Fig. 2e–h) characteristics, suggesting that it is a consistent empirical feature of urban supply chains. For example, the SCI is positively related to population (Fig. 2a and Supplementary Table 1; slope = 0.8, *R*^*2*^ = 0.20, *P* < 0.001) whereas SCI′ shows a negative dependency on population (Fig. 2a and Supplementary Table 1; slope = -0.43, *R*^*2*^ = 0.09, *P* = 0.012). This indicates that a city’s shares of supply inflows become more concentrated in high-complexity products as city size (population) increases, whereas its shares of supplier connections increases for low-complexity products. This interaction strength pattern is consistent with the observation that the complexity of urban economic activity rises with city size^27^, which for large cities would imply an increase in the complexity of supply chains, assuming that more complex supply inflows require more component parts and supply chain stages^19^.

The distribution of the number of suppliers in a city’s imports is an important constraint on the observed pattern for SCI′ (Fig. 2). To meet urban demand for low-complexity products, which tend to be associated with resource-constrained products (e.g., agricultural and mining products)^23^, large cities require a greater share of supplier connections than medium-sized cities. Taking the share of supplier connections as a measure of suppliers’ relative diversity^8,9,12^, the suppliers’ relative diversity for low-complexity products tends to increase with city size, which makes the SCI′ decline (Fig. 2a and Supplementary Table 1). In addition, the linear regression fits between supply chain complexity and local supply network characteristics are better for SCI′ than for SCI (Fig. 2e–h), suggesting that SCI′ is more effective than SCI at differentiating city supply network structure.

### Association between supply chain shock intensity and supply chain complexity

**Fig. 3:**
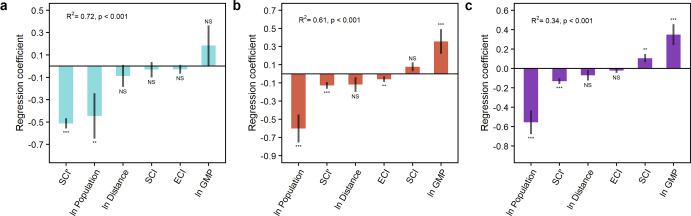
Effect of supply chain complexity on the intensity of supply chain shock. Coefficients associated with the regression of the supply chain shock intensity on the supply chain complexity indices and several control variables [ln population, ln average distance, standardized economic complexity index (ECI), and ln gross metropolitan product (GMP)]. The shock intensities are calculated using data for 2012-2015 and the explanatory variables using data for 2012. **a** Coefficients obtained using the average shock intensity of a city’s main imports as the response variable (*n* = 69). **b** Coefficients obtained using the average shock intensity of all imports to a city as the response variable (*n* = 69). (**c**) Coefficients obtained using the shock intensity of individual city-product pairs as the response variable (*n* = 2512). The standardized regression coefficients are shown in Fig. S6. ***, *P* < 1%; **, *P* < 5%; and NS (nonsignificant), *P* > 10%. The standard error is indicated with the black vertical line. These regression analyses are for the city data only.

Using nested, cross-sectional regression models, we find that the supply chain shock intensity experienced by cities strongly decreases with a city’s horizontal supply chain complexity (Fig. 3 and Supplementary Fig. 5). This finding holds up after accounting for a variety of model specifications (Supplementary Tables 3–10). The shock intensity is calculated for each product-region pair as the largest negative, annual inflow deviation from the average inflow during 2012–2015 (Methods)^10^. With shock intensities varying from ~1% to 85% (Supplementary Fig. 4), supply chain shocks during 2012–2015 cover a wide range of cases for evaluating the association between supply chain shocks and supply chain complexity.

For the first set of nested regression models (Supplementary Tables 1 and 2), we relate the supply chain complexity indices to the average shock intensity of a city’s main imports. This model explains 72% of the variation in the average shock intensity across cities (Fig. 3a, Supplementary Fig. 7, and Supplementary Table 3). Both urban population and SCI′ are strongly and negatively associated with the average shock intensity (Fig. 3a), suggesting that large cities as well as cities with high horizontal supply chain complexity tend to experience less intense shocks, on average.

Letting reductions in the average shock intensity across cities be indicative of a greater resistance to shocks, both the local (population) and network (SCI′) effects influence a city’s resistance to supply chain shocks. Resistance, defined as the ability to buffer or avoid shocks, is a key aspect of resilience^28^. Given that large cities tend to have low SCI′ values (Fig. 2a), they benefit from the local effect, whereas the network effect is important to medium-sized cities. A possible mechanism for this local effect is the weaker dependence of large cities on supply networks, at least in the sense that a larger share of their trade is local^23,27^, which may serve to dissipate small to moderate supply chain shocks^29^.

### Robustness of our findings

To further evaluate the sensitivity of our results, we implement two other sets of nested regression models. For the second set of models (Fig. 3b, Supplementary Fig. 8, and Supplementary Table 4), the specifications are the same as in the first set, except that a city’s shock intensity is calculated as the average shock from all its imports. The results from this analysis reinforce our finding that both urban population and SCI′ have a significant negative association with shock intensity. For example, a large 10% increase in population reduces the average shock intensity by 6%, whereas an increase of one standard deviation in SCI′ reduces the average shock intensity by ~13% (Fig. 3b and Supplementary Table 4). Overall, the results are similar when employing standardized coefficients (Supplementary Fig. 6). Although in our analyses the association between the average shock intensity and SCI′ is strong (*P* < 0.01; Fig. 3a, b), care is needed in interpreting these results as causal, because only cross-sectional analyses can be performed due to the relatively short duration of the available supply network data.

For the third set of nested regression models (Fig. 3c and Supplementary Tables 5, 6), the results are consistent with our previous models, even though this third set uses the shock intensity of individual product-region pairs as the response variable, resulting in a much larger sample size (*n* = 2512). For instance, a large 10% increase in population reduces the shock intensity by 5.6%, whereas a one standard deviation increase in SCI′ reduces the shock intensity by ~13% (Fig. 3c and Supplementary Table 5). For this analysis, SCI has a slightly significant and positive effect on the shock intensity (Fig. 3c), indicating that the influence of horizontal and vertical complexity on supply chain shocks may differ. Overall, we find similar results when considering control variables other than those in Fig. 3c (Supplementary Tables 7–10). For the third analysis, however, the explained variation is less (Fig. 3c) than in the first and second regression analyses (Fig. 3a, b), suggesting that our approach is better at predicting a city’s shock intensity across multiple products than individual ones. Despite the drop in the explained variation, the coefficient for SCI′ is consistently negative and strongly significant across all the regression analyses (Fig. 3 and Supplementary Tables 3 to 10).

We perform two additional robustness checks to test the influence of our shock intensity measure (Eq. 8) on the regression analyses. For the first robustness check, given that Eq. 8 is most useful for stationary time series data, we recalculate the regression analyses using stationary data alone, by removing from the dataset the time series for individual product-region pairs with significant linear trends at a 5% significance level (25% of the data). The results from this analysis (not shown) are consistent with our regression findings based on the entire dataset (Fig. 3). For the second robustness check, we use the average fluctuation of supply inflows instead of shock intensity (Methods) as the response variable in the regression analyses. For this analysis, we find that *R*^*2*^ values improve slightly compared to the values reported in Fig. 3. In addition, the coefficient for SCI′ is negative and significant (*P* < 0.01), indicating that SCI′ values tend to increase as the average fluctuation of supply inflows declines or supply chain stability improves. Taking gains in supply chain stability as indicative of resilience, this result is consistent with our key finding that a city’s resilience to supply chain shocks increases with SCI′, on average.

### Outlook

Across our 69 cities, the supply network and local city effects complement each other in reducing the supply chain shock intensity (Fig. 3). A central tenant of urban economic theory is that urban economic benefits are offset by costs^30^, which leads to spatial equilibrium and ultimately long-run stability in an urban system of interacting cities and regions^30,31^. Extending this idea to our results, and assuming that costs will tend to increase for cities that experience higher-intensity shocks, both network and local city effects may be seen as stabilizing forces in the supply chains of cities. Specifically, the network effect may be crucial for reducing shock intensity in medium-sized cities (Fig. 3), which could be used to help manage supply chain risks, for example, by facilitating gains in horizontal supply chain complexity.

To quantify and visualize the relationship between the risk of supply chain shock and horizontal supply chain complexity, one can plot the probability of cities experiencing a supply shock greater than a certain threshold against their average SCI′ value (Supplementary Fig. 5b). Such graphical tools could help cities in managing their supply chain risks, as highlighted by Gomez et al.^10^. For the food sector in the United States, Gomez et al.^10^ found that increasing functional supply chain diversity can reduce the probability of food supply shock to cities. To reduce the risk of supply shock to cities in a multisector supply network, our results indicate that improvements in supply chain diversity (horizontal supply chain complexity) in one sector may need to be balanced against improvements in other sectors. Our findings point to the need for a holistic, multisector approach to supply chain design and policy.

The COVID-19 pandemic has exposed vulnerabilities in the global supply chains. In response, governments and companies have taken action to try to enhance supply chain resiliency^32^. Our results highlight the possibility of boosting resiliency through coordinated actions that foster supplier diversity for high-complexity products in urban supply chains. Our data-driven approach is general and potentially applicable at different levels of analysis, from individual companies to countries. This, coupled with emerging datasets from smart technologies^33^, could make our results relevant beyond cities to a wide spectrum of supply chain actors.

## Methods

### Supply chain networks

We use annual commodity flow networks to represent supply chains in the United States during 2012–2015. The networks are obtained from the Freight Analysis Framework version 4 (FAF4) database^25^. The FAF4 provides empirical, annual commodity flow data for the year 2012, and annual reanalysis data for the years 2013–2015. All data are in units of tons per year or United States dollars per year. The reanalysis data are obtained by combining a national macroeconomic model, regional trade modeling, and fine-grained empirical economic data^34^.

The FAF4 database divides the contiguous United States into 132 domestic regions and the rest of the world into eight international regions. For the United States, the domestic regions consist of 85 metropolitan statistical areas or cities, 35 remainders of states, and 12 states. The remainder of a state is the area of a state that is not part of one of the FAF4 cities. For example, the FAF4 includes the cities of Philadelphia and Pittsburgh in the state of Pennsylvania. The remainder of Pennsylvania is the area of the state not covered by these two cities. The state regions in FAF4 are states without any FAF4 cities in them; each of these states represents a single region. Further, some of the FAF4 cities are broken down into parts based on state lines. For example, Philadelphia is divided into four areas since its metro area falls under four different states—Delaware, Maryland, New Jersey, and Pennsylvania. For each city that is divided into parts, we combine the parts into a single unit. This reduces the number of domestic FAF4 cities from 85 to 69 and the total number of domestic FAF4 regions from 132 to 115 (Fig. 1, f and g, illustrate the 115 domestic regions). To calculate the supply chain complexity indices, we use these 115 domestic regions and the 8 international FAF4 regions—Canada, Mexico, Rest of Americas, Europe, Africa, Southwest and Central Asia, Eastern Asia, and Southeast Asia & Oceania.

Using 41 different product categories, the FAF4 database covers the entire product economy of the United States^25^. For our analysis, we use 39 out of these 41 product categories, leaving out only the product categories for “mix of unclassified flows” and “waste.” Since the FAF4 data consist of annual origin-destination flows between geographic location pairs, the data can be visualized as spatial supply networks, with links representing the value of supply flows. When interpreted in this way, the FAF4 database consists of a unique supply network for each product category in a given year, with all networks sharing the same 123 nodes (115 domestic nodes and 8 international nodes). Thus, for our analyses, we use a total of 156 supply networks (39 product categories × 4 years of data = 156 supply networks) that together represent more than 1 million annual supply flows. Figure 1a illustrates the domestic supply network for pharmaceutical products.

### Supply chain complexity indices

When estimating supply chain complexity, we distinguish between upstream horizontal (SCI′) and vertical (SCI) supply chain complexity, which are considered key structural dimensions of supply chains in supply chain theory and management^4,35–37^. Hereafter SCI is exclusively used to indicate vertical complexity and SCI′ to indicate horizontal complexity. *Vertical supply chain complexity* refers to the upstream depth of supply chains, whereas *horizontal supply chain complexity* refers to the number of immediate upstream connections or suppliers^4,37–40^. To account for these dimensions with our supply networks, we use the in-strength (i.e., a product’s total inflow to a region) and in-degree (i.e., a region’s total number of supplier connections for a given product) of a node (region) to quantify the vertical and horizontal complexity, respectively. For a given product or commodity type, the total inflow to a region is equal to the sum of the supply flows from all other regions, whereas the total number of supplier connections is equal to the number of different regions that supply a given product or commodity to a region.

We assume that a product’s level of technological sophistication or complexity reflects its upstream supply chain complexity. This is a reasonable assumption because more complex products tend to have more component parts and require more supply chain stages for production^19,24,41^. To rank the complexity of our 39 product categories, we use the eigenvalue approach of Mealy et al. ^20^, which is equivalent to the method of reflections of Hidalgo and Hausmann^22^. This way of ranking product complexity has been shown to work well at different levels of product and spatial aggregation^23,27,42–48^, including metropolitan areas or cities in the United States^23^. The approach is essentially a dimensionality reduction algorithm that extracts or learns key patterns from the data^21^. When applied to commodity flows or city-level economic data^23,27,42–48^, the algorithm has been shown to successfully classify the complexity of products or economic activity and to distinguish between regional economic specialization patterns. This is in part the reason why we use the algorithm here to derive our supply chain complexity indices.

To calculate the SCI and SCI′, we aggregate the spatial supply networks for a given year into a single, unweighted and undirected, product-region bipartite network with dimensions of 115 × 39 (Fig. 1b). The two groups of nodes in the bipartite network consist of the 39 product categories linking to the 115 domestic regions in the FAF4 database (Fig. 1b). The links on the bipartite network are assigned using a LQ equation^20,23,49^. Using the total supply inflows of product *p* to region *r*, $x_{rp}$, we obtain LQ for each product-region pair as follows:

1 $$LQ_{rp} = \frac{{x_{rp}/\mathop {\sum }\nolimits_p x_{rp}}}{{\mathop {\sum }\nolimits_r x_{rp}/\mathop {\sum }\nolimits_r \mathop {\sum }\nolimits_p x_{rp}}}$$

For each product category, $LQ_{rp}$ measures the concentration of a region’s supply inflows (numerator of Eq. (1) relative to the product’s national concentration (denominator of Eq. (1). Note that to calculate SCI we use the region’s share of inflow products in Eq. (1), whereas for SCI′ we use the region’s shares of supplier connections.

With the following condition, we emphasize a region’s most dominant supply inflows

2 $$M_{rp} = \left\{ {\begin{array}{*{20}{l}} {0,\;LQ_{rp} < 1} \\ {1,\;LQ_{rp} \ge 1} \end{array}} \right.$$

This results in a product-region bipartite network defined by the binary matrix *M* with elements $M_{rp}$ and links assigned to product-region pairs with $LQ_{rp} \ge 1$. The *LQ* threshold of 1 allows us to focus on the supply chains that make a region unique or different from other regions. This is useful here because in our dataset every region requires inputs from all the different product categories. In addition, for low (e.g., $LQ_{rp} \ge 0.5$) or high (e.g., $LQ_{rp} \ge 1.5$) values of *LQ*, the value of the supply chain complexity index is similar across cities (Supplementary Fig. 1), so *LQ* values between 0.5 and 1.5 are desirable. Ultimately, we select $LQ_{rp} \ge 1$ because this value captures differences or heterogeneity in the supply chain complexity indices across our 69 cities (Supplementary Fig. 1), and it has been used before to study the complexity of cities^20,23^.

The column-wise and row-wise sums of *M* give the diversity of regions *d* and the ubiquity of products *u*^20,22^, respectively. The elements of *d* and *u* are given by

3 $$d_r = \mathop {\sum}\limits_p {M_{rp}}$$

4 $$u_p = \mathop {\sum}\limits_r {M_{rp}}$$

The variables in Eqs. 2–4 can be used to determine the matrices $\tilde M$ and $\hat M$ that capture the similarity between the supply chain structures of regions^20^. $\tilde M$ captures the similarity between supplies—similarity weighted by the regions’ diversity—and $\hat M$ the similarity between suppliers—similarity weighted by the products’ ubiquity.

5 $$\widetilde M = D^{ - 1}MU^{ - 1}M^{\prime}$$

6 $$\widehat M = U^{ - 1}M\prime D^{ - 1}M$$

where *D* and *U* are the diagonalized *d* and *u* matrices, respectively, and *M* is the binary product-region matrix. Following the eigenvalue approach of Mealy et al.^20^, SCI is defined as the eigenvector associated with the second largest right eigenvalue of $\tilde M$, while the ranking of product complexity is defined by the eigenvector associated with the second largest right eigenvalue of $\hat M$. Note that the ranking of product complexity is typically referred to as the product complexity index^20,22^. Moreover, Eqs. (1–6) are used to calculate both SCI and SCI′, with the only difference being that for SCI $x_{rp}$ is equal to the share of a region’s inflows for a given product *p* and for SCI′, it is equal to the share of a region’s number of supplier connections for a given product *p*.

Alternatively, the matrix $\tilde M$ (Eq. 5) can be expressed as follows:

7 $$\widetilde M = D^{ - 1}K\;{{{\mathrm{where}}}}\;K = MU^{ - 1}M^\prime$$

*K* is the symmetric similarity matrix, which places cities with similar supply chain structure closer together and cities with dissimilar structure farther apart (Fig. 1c, d). In addition, Eq. (7) allows interpretation of the algorithm as spectral clustering^20^. Following this interpretation, the algorithm aims to partition regions into two main supply chain structure groups at a value of SCI or SCI′ equal to 0 (Fig. 1e).

### Supply chain shocks

We estimate supply chain shocks to investigate the ability of cities to avoid or resist shocks. Resistance, together with recovery time and robustness, is one of the three main components of resilience^28,50,51^. To calculate the supply chain shock intensity for each product-region pair, we use the supply network data during 2012–2015^10,52^. For each region *r* and product *p*, we calculate the supply chain shock intensity $S_{rp}$ as follows:

8 $$S_{rp} = \left[ {1 - \frac{{\min \left( {I_r^p} \right)}}{{\left\langle {I_r^p} \right\rangle }}} \right] \times 100$$

where $I_r^p$ is the time series of total inflows to node *r* for product *p* for 2012–2015, and min($I_r^p$) and $\left\langle {I_r^p} \right\rangle$ are the minimum and average values of the time series $I_r^p$, respectively. We use the supply chain shock intensities determined with Eq. (8) as the response variable in the regression analyses. Equation (8) is applicable to our dataset since most of the times series $I_r^p$ (75% of the data) do not exhibit a significant linear trend at a 5% significance level. Nonetheless, we test this assumption as part of our regression analyses.

In addition, we determine the probability of shock for a subset *S* of all $S_{rp}$ shocks. To do this, we divide the supply chain complexity of cities into *b* bins. For each bin *b*, we count the number of observations $n_b$ that meet the criteria *S* > *s* for *s* $\in$ {5, 10, 15, 20}, with *s* being the shock intensity threshold. The probability of a supply shock being greater than *s* in bin *b* is calculated as follows:

9 $$P_b(S \,>\, s) = \left[ {1 - \frac{{n_b}}{{N_b}}} \right]$$

where *N*_*b*_ is the total number of observations in bin *b*. Thus, for each shock intensity *s*, we obtain a set of probabilities of supply chain shock, $P\left( {S \,>\, s} \right) = P_b(S \,>\, s)$ for *b* = {1, …, 5}. We use these probabilities to plot the relationship between the probability of supply chain shock and supply chain complexity.

To overcome possible limitations with our shock measure (Eq. 8), we evaluate using the average fluctuation of supply inflows as an alternative to Eq. (8). For each times series $I_r^p$, the fluctuation value is calculated as the average of the absolute difference of supply inflows between consecutive years. That is, for the time series associated with each product-region pair, we average the absolute difference between supply inflows in 2012–2013, 2013–2104, and 2014–2015. This way of calculating fluctuations avoids having to estimate higher-order statistics, which can be challenging with our short time series data.

### Statistical analyses

We use ordinary least squares multiple linear regression to assess the association between supply chain shock intensity and supply chain complexity with the natural logarithm of the shock intensity as the response variable. When using the average shock intensity of a city’s imports as the response variable, the regression equation has the following form:

10 $$\ln (\hat S_r) = b_0 + b_1SCI_r + b_2SCI_r^\prime + b_3SCI_r \times SCI_r^\prime + \mathop {\sum}\limits_{i > 3} {b_i} \ln \left( {x_{ir}} \right) + \varepsilon _r$$

where $\hat S_r$ is the average supply chain shock intensity for city *r*; $SCI_r$ and $SCI_r^\prime$ are the vertical and horizontal supply chain complexity indices for city *r*, respectively; the term $x_{ir}$ represents a control variable *i* (e.g., population, gross metropolitan product, or average shipment distance); and $\varepsilon _r$ is the error term. The *b*_*i*_ terms are the regression coefficients. When using the shock intensity of individual products as the response variable, we use the following regression equation:

11 $$\begin{array}{l}\ln (\hat S_{rp}) = b_0 + b_1SCI_r + b_2SCI_r^\prime + b_3SCI_r \times SCI_r^\prime\\\qquad\qquad\; +\, \mathop {\sum}\limits_{i > 3}^N {b_i} \ln \left( {x_{ir}} \right) + \mathop {\sum}\limits_{i > N} {b_i} \ln \left( {x_{irp}} \right) + \varepsilon _{rp}\end{array}$$

where $\hat S_{rp}$ is the supply chain shock intensity and $x_{irp}$ is a control variable *i* for city *r* and product *p*. All other variables and coefficients in Eq. (11) have the same meaning as in Eq. (10). Further details about the regression models are included in the Supplementary Methods.

We perform four different regression analyses. In the first analysis (I), the average shock intensity of the main inflow products to a city (i.e., product-region pairs with $LQ_{rp} \ge 1$) is used as the response variable (*n* = 69 cities). In the second analysis (II), the average shock intensity of all inflow products to a city is used as the response variable (*n* = 69 cities). In the third (III) and fourth (IV) regression analyses, the individual shocks associated with all possible product-region pairs is used as the response variable (*n* = 2513 product-region pairs). The difference between analyses III and IV is that in analysis IV we consider additional control variables than in analysis III. These four regression analyses are designed to progressively increase the robustness and generality of our results.

For analyses I–III, we use four different control variables for the year 2012. The variables include urban population (number of persons)^53^, gross metropolitan product (GMP) (US$)^54^, economic complexity index (ECI)^20–22^, and the average shipment distance (miles)^25^. In addition, for analysis III, we include dummy variables for each of the product categories. These control variables were selected based on previous studies^4,55,56^, which have shown that size, competitiveness, specialization, shipment distance, and type of product are key factors that influence supply chain complexity and shock intensity.

We use urban population to measure city size^13,15,57–60^. More generally, urban population is used to account for cities’ local characteristics since it strongly relates to many social, economic, and infrastructural properties of cities that tend to scale as a universal power-law relationship of city size^13,59^. Shipment distance is often considered as a proxy for transportation costs^3,61–63^. For a given product, the average shipment distance is calculated as the average shipment distance of all inflows to a city, which for a given city results in an average distance for each of the 39 product categories in our dataset. To obtain a single distance value for a city, we further average the distances across the city’s inflow product categories. For each commodity flow in the supply networks, the shipment distance is obtained from the FAF4 database^25^. We use GMP or the level of economic activity in each city as a proxy for urban competitiveness and the ECI to quantify economic specialization^20,23^. In addition, ECI has been used to measure urban economic diversity^23,64,65^ and predict urban economic growth^23,65^ in United States cities. Note that we use Eqs. (1–6) to calculate ECI. However, differently from our calculation of SCI, we let $x_{rp}$ in Eq. (1) be equal to a region’s share of outflows for a given product when determining ECI.

We use regression analysis IV to further evaluate the sensitivity of our results. This analysis has two parts, IV.a and IV.b, which only differ in the year used to calculate the supply chain complexity indices. For analysis IV.a, the supply chain complexity indices are estimated using annual supply network data for the year 2012, whereas for analysis IV.b data for 2015 are used. This is done to assess the sensitivity of our results to the year used to calculate the indices. For low and high values of the supply chain complexity indices, the indices tend to remain fairly constant from 2012 to 2015. For intermediate values, the indices tend to vary across years for some of the cities in our dataset (Supplementary Fig. 3).

Furthermore, we consider in analyses IV.a and IV.b three additional control variables, including the percent of foreign-sourced supplies, the percent of urban-sourced supplies, and the total production of products (US$). The percent of foreign-sourced supplies is the share of the total inflows of product *p* to region *r* sourced from the eight FAF4 international regions. This variable accounts for possible differences between products sourced domestically versus internationally^66,67^. The percent of urban-sourced supplies is the share of the total inflows of product *p* to region *r* sourced from other FAF4 cities. This variable accounts for possible differences between intercity and non-intercity interactions^14,15,68^. For each product-region pair, a city’s total production is calculated as the node’s out-strength based on the aggregation of all the FAF4 supply networks. This variable is useful because, differently from GMP, it solely accounts for products, excluding services that take on a large share of GMP. These three control variables are estimated using the FAF4 data for the year 2012.

Prior to the regression analyses, we calculate the variance inflation factors for all the predictor variables (Supplementary Table 2). For all the regression models, we perform diagnostic checks for homoscedasticity/non-autocorrelation, normality, and leverage and influential points (Supplementary Figs. 7–11)^69^.

## Supplementary information

Supplementary Material

nr-reporting-summary

Data 1

## Data Availability

All data are available in the main text or the supplementary information.

## Code availability

The codes used in this study are available from the corresponding author upon reasonable request.
