# Supplementary material for: Cities can benefit from complex supply chains
Source: NPJ Urban Sustain. 2023 Mar 29;3(1):20. doi: 10.1038/s42949-023-00100-5 (PMC10052311; doi:10.1038/s42949-023-00100-5)
Supplement: Supplementary file 1 — Supplementary Material [file 42949_2023_100_MOESM1_ESM.pdf]

Supplementary Information

Cities Can Benefit from Complex Supply Chains

Nazlı B. Doğan, Alfonso Mejia, and Michael Gomez

Correspondence to: aim127@psu.edu

This PDF file includes:

Supplementary Methods
Supplementary Figures 1 to 11
Supplementary Tables 1 to 10

Other Supplementary Information for this manuscript include the following:

Data 1 Dataset used in the regression analyses (separate file)

Supplementary Methods

Detailed Description of the Regression Models

For regression analysis I, we use the following multivariate, multiple linear regression models:

Model 1:
$$\ln(\hat{S}_r^v, \hat{S}_r^h) = b_0 + b_1 \ln(p_r) + b_2 \ln(GMP_r) + b_3 ECI_r + b_4 \ln(d_r) + b_5 SCI_r + b_6 SCI'_r + \varepsilon_r \quad (1)$$

Model 2:
$$\ln(\hat{S}_r^v, \hat{S}_r^h) = b_0 + b_1 \ln(p_r) + b_2 \ln(GMP_r) + b_3 ECI_r + b_4 \ln(d_r) + \varepsilon_r \quad (2)$$

Model 3:
$$\ln(\hat{S}_r^v, \hat{S}_r^h) = b_0 + b_1 \ln(p_r) + b_2 \ln(GMP_r) + b_3 ECI_r + b_4 \ln(d_r) + b_5 SCI_r + b_6 SCI'_r + b_7 SCI_r \times SCI'_r + \varepsilon_r \quad (3)$$

where $\hat{S}_r^v$ and $\hat{S}_r^h$ are the average supply chain shocks associated with SCI and SCI′, respectively, for city r; p_r is the population of city r; GMP_r is the gross metropolitan product of city r; d_r is the average shipment distance of products delivered to city r; and ε_r is the error term. The b_i terms are the regression coefficients. Note that the average shock intensity of city r differs between SCI and SCI′. This is because for this first analysis the average shock intensity is calculated based on the region-product pairs with $LQ_{rp} \geq 1$, which varies between SCI and SCI′. Model 1 (Eq. 1) considers both control and main effect variables, while model 2 (Eq. 2) only considers control variables. The purpose of model 2 is to help isolate the influence of SCI and SCI′ on the response variable and model performance. Model 3 (Eq. 3) accounts for a possible interaction effect between SCI and SCI′, but otherwise the model is similar to Eq. 1.

For regression analysis II, we use as the response variable the average shock intensity $\hat{S}_r$, calculated as the average shock across all inflow products to city r. For this analysis, the following three regression models are used:

Model 1:
$$\ln(\hat{S}_r) = b_0 + b_1 \ln(p_r) + b_2 \ln(GMP_r) + b_3 ECI_r + b_4 \ln(d_r) + b_5 SCI_r + + b_6 SCI'_r + \varepsilon_r \quad (4)$$

Model 2:
$$\ln(\hat{S}_r) = b_0 + b_1 \ln(p_r) + b_2 \ln(GMP_r) + b_3 ECI_r + b_4 \ln(d_r) + \varepsilon_r \quad (5)$$

Model 3:
$$\ln(\hat{S}_r) = b_0 + b_1 \ln(p_r) + b_2 \ln(GMP_r) + b_3 ECI_r + b_4 \ln(d_r) + b_5 SCI_r + b_6 SCI'_r + b_7 SCI_r \times SCI'_r + \varepsilon_r \quad (6)$$

The definition of the variables and coefficients in Eqs. 4-6 is the same as in Eqs. 1-3.

For regression analysis III, the individual shocks associated with all possible region-product pairs are used as the response variable. Besides the two main effect variables, SCI and SCI′, and the four control variables used in the previous regression analyses, this analysis includes dummy variables for each product category. A total of 36 dummy variables are used, each coded as a binary or indicator variable with “alcoholic beverages” as the baseline product. These dummy variables account for differences across products. The following three models are used for this analysis:

Model 1:

$$\ln(\hat{S}_{rp}) = b_0 + b_1 \ln(p_r) + b_2 \ln(GMP_r) + b_3 ECI_r + b_4 \ln(d_{rp}) + \sum_{p=1}^{36} b_{5,p} Industry_{rp} + b_6 SCI_r + b_7 SCI'_r + \varepsilon_{rp} \quad (7)$$

Model 2:

$$\ln(\hat{S}_{rp}) = b_0 + b_1 \ln(p_r) + b_2 \ln(GMP_r) + b_3 ECI_r + b_4 \ln(d_{rp}) + \sum_{p=1}^{36} b_{5,p} Industry_{rp} + \varepsilon_{rp} \quad (8)$$

Model 3:

$$\ln(\hat{S}_{rp}) = b_0 + b_1 \ln(p_r) + b_2 \ln(GMP_r) + b_3 ECI_r + b_4 \ln(d_{rp}) + \sum_{p=1}^{36} b_{5,p} Industry_{rp} + b_6 SCI_r + b_7 SCI'_r + b_8 SCI_r \times SCI'_r + \varepsilon_{rp} \quad (9)$$

The variables in Eqs. 7-9 are the same as in analyses (I) and (II), with the exception of d_{rp}, which is the average shipment distance travelled by product p to region r; $Industry_{rp}$, which is a categorical variable representing the name of product p supplied to region r; and the error term ε_{rp}. Model 1 (Eq. 7) includes the control and main effect variables, model 2 (Eq. 8) includes only the control variables, and model 3 (Eq. 9) incorporates the interaction effect.

Regression analysis IV is similar to analysis III, except that it includes three additional control variables, u_{rp}, f_{rp}, and o_{rp}. Also, recall that analysis IV is done using supply chain complexity indices calculated using data for 2012 (analysis IV.a) and for 2015 (analysis IV.b). Since analyses IV.a and IV.b are otherwise the same, Eqs. 10-12 below apply to both analyses. Using the individual shocks associated with all possible region-product pairs as the response variable, the following three models are evaluated:

Model 1:

$$\ln(\hat{S}_{rp}) = b_0 + b_1 \ln(p_r) + b_2 \ln(GMP_r) + b_3 ECI_r + b_4 \ln(d_{rp}) + b_5 f_{rp} + b_6 u_{rp} + b_7 \ln(o_{rp}) + \sum_{p=1}^{36} b_{8,p} Industry_{rp} + b_9 SCI_r + b_{10} SCI'_r + \varepsilon_{rp} \quad (10)$$

Model 2:

$$\ln(\hat{S}_{rp}) = b_0 + b_1 \ln(p_r) + b_2 \ln(GMP_r) + b_3 ECI_r + b_4 \ln(d_{rp}) + b_5 f_{rp} + b_6 u_{rp} + b_7 \ln(o_{rp}) + \sum_{p=1}^{36} b_{8,p} Industry_{rp} + \varepsilon_{rp} \quad (11)$$

Model 3:

$$\ln(\hat{S}_{rp}) = b_0 + b_1 \ln(p_r) + b_2 \ln(GMP_r) + b_3 ECI_r + b_4 \ln(d_{rp}) + b_5 f_{rp} + b_6 u_{rp} + b_7 \ln(o_{rp}) + \sum_{p=1}^{36} b_{8,p} Industry_{rp} + b_9 SCI_r + b_{10} SCI'_r + b_{11} SCI_r \times SCI'_r + \varepsilon_{rp} \quad (12)$$

The variables in Eqs. 10-12 are the same as those in Eqs. 7-9, with the exception of u_{rp}, f_{rp}, and o_{rp}. f_{rp} is the percent of foreign-sourced supplies of product p to city r, u_{rp} is the percent of urban-sourced supplies of product p to region r, and o_{rp} is the total production of product p in region r. As in the previous analyses, model 1 (Eq. 10) includes the control and main effect

variables, model 2 (Eq. 11) includes only the control variables, and model 3 (Eq. 12) incorporates the interaction effect.

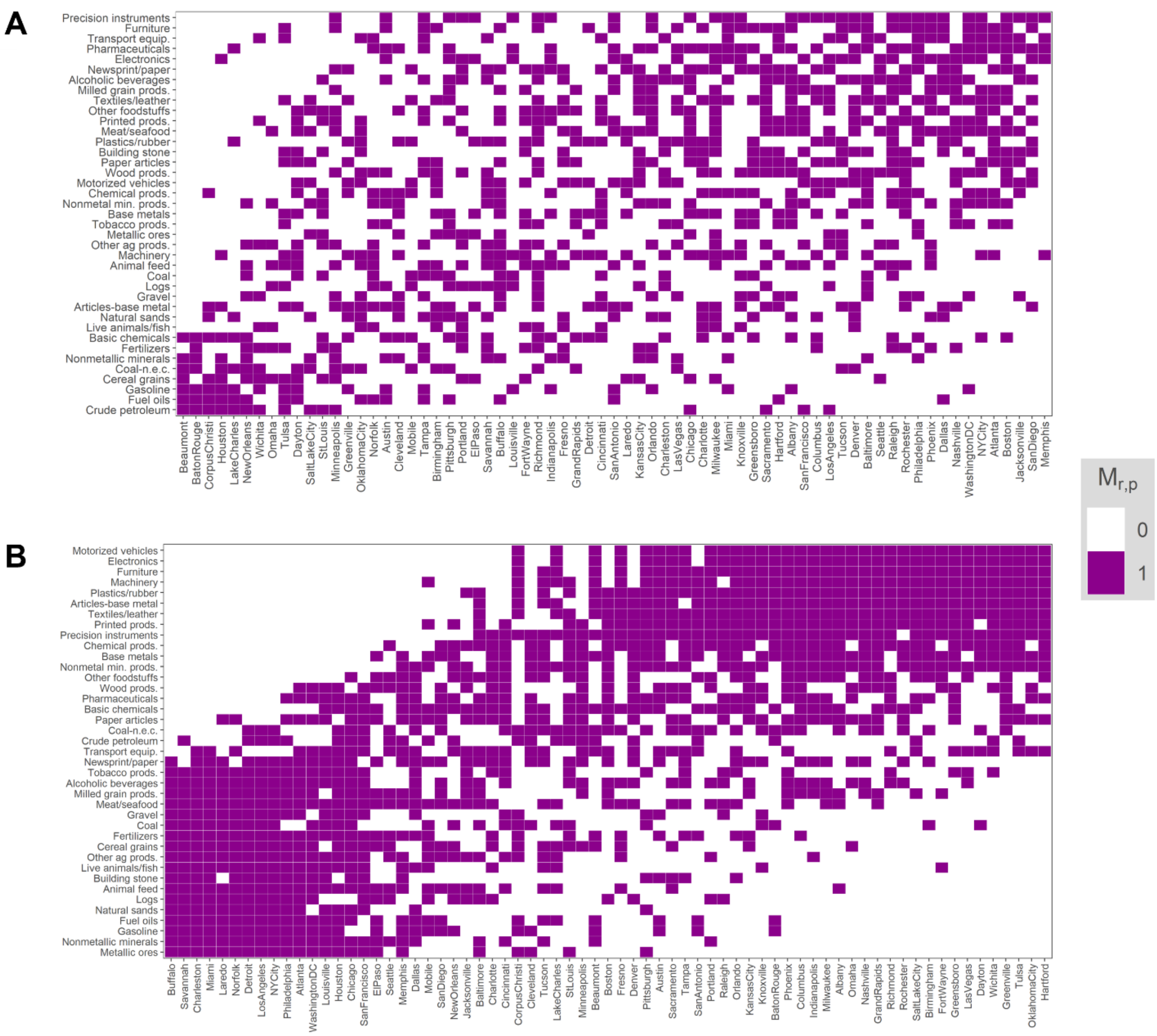

Supplementary Figure 1.

Binary product-region matrix used to calculate SCI (A) and SCI′ (B) (only cities are shown). Products (*y* axis) and regions (*x* axis) are ranked in ascending order according to their product complexity values and SCI (or SCI′), respectively. Equip., equipment; prods., products; min., mining; ag., agricultural; n.e.c., not elsewhere classified. SCI, vertical supply chain complexity index; SCI′, horizontal supply chain complexity index.

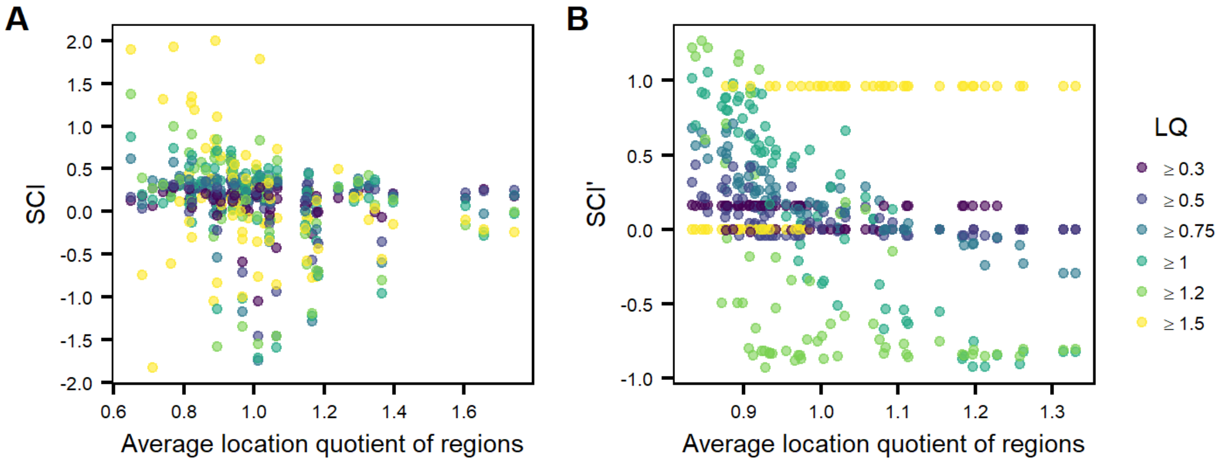

Supplementary Figure 2.

Average location quotient (LQ) of regions for different LQ thresholds (0.3, 0.5, 0.75, 1, 1.2, 1.5) versus the SCI (A) and SCI′ (B). SCI, vertical supply chain complexity index; SCI′, horizontal supply chain complexity index.

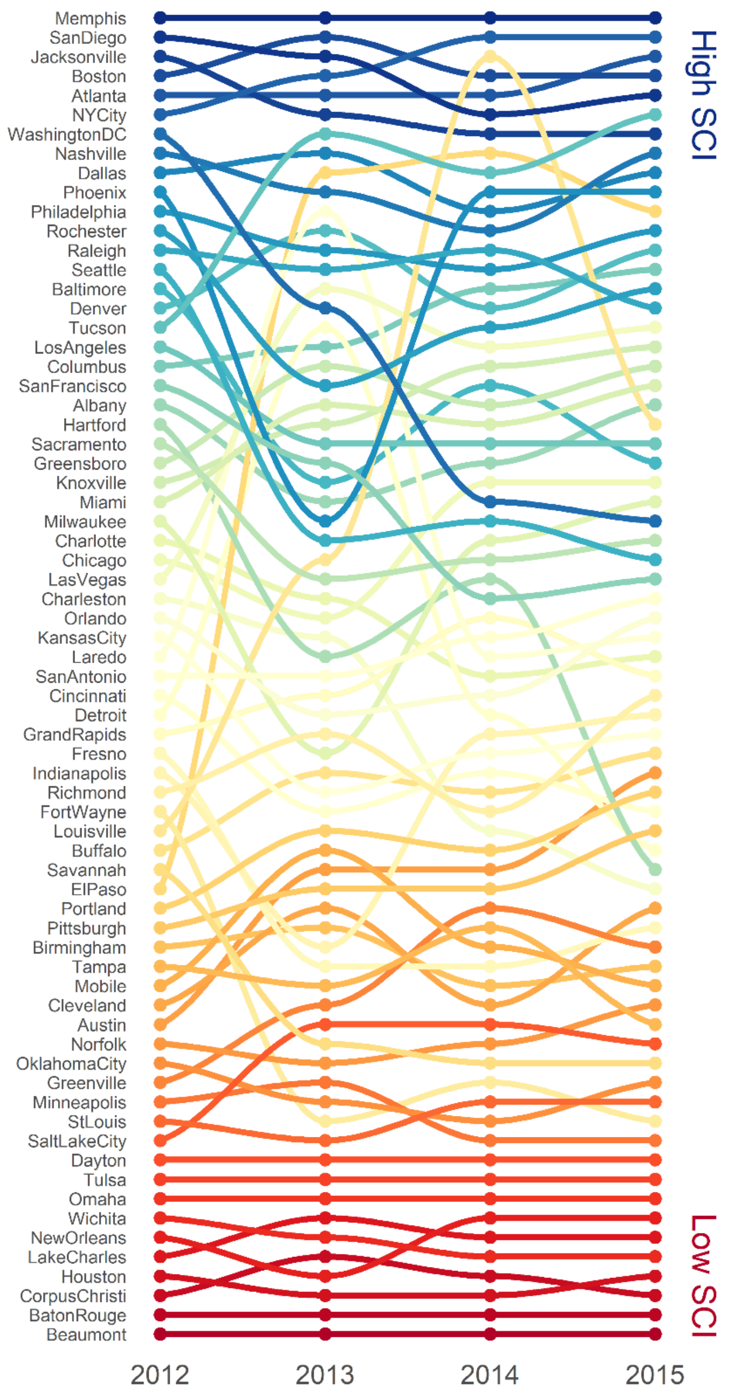

Supplementary Figure 3.

Annual time series of SCI values for our 69 cities from 2012-2015. The ranking of cities with low and high SCI values remained fairly stable across the years. SCI values tended to vary for some medium-sized cities, such as El Paso, TX, Louisville, KY, and Hartford, CT.

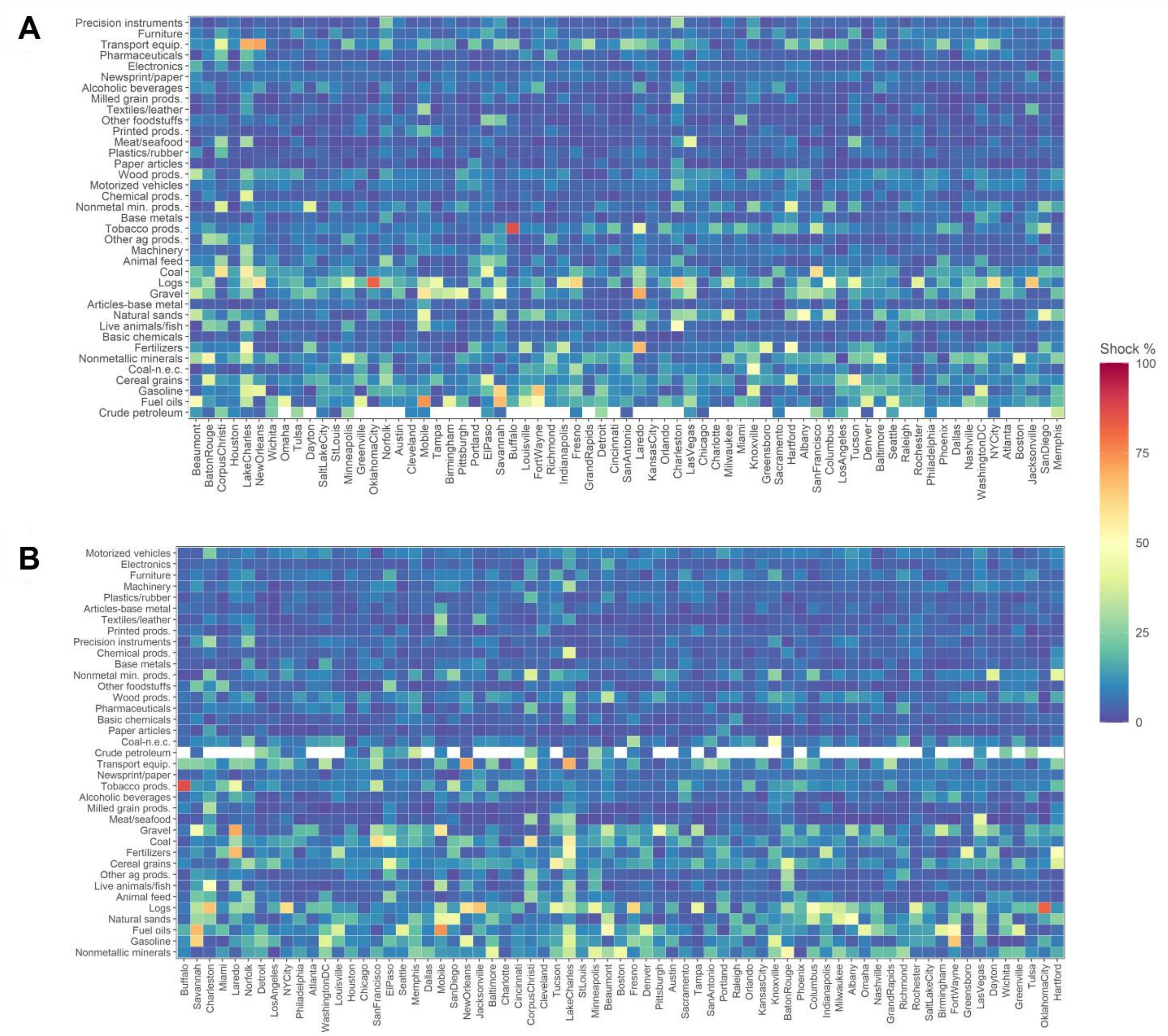

Supplementary Figure 4.

Shock intensity values for product-city pairs ranked in ascending order according to their product complexity values and SCI (A) or SCI′ (B). The blank cells for crude petroleum indicate missing values. Equip., equipment; prods., products; min., mining; ag., agricultural; n.e.c., not elsewhere classified. SCI, vertical supply chain complexity index; SCI′, horizontal supply chain complexity index.

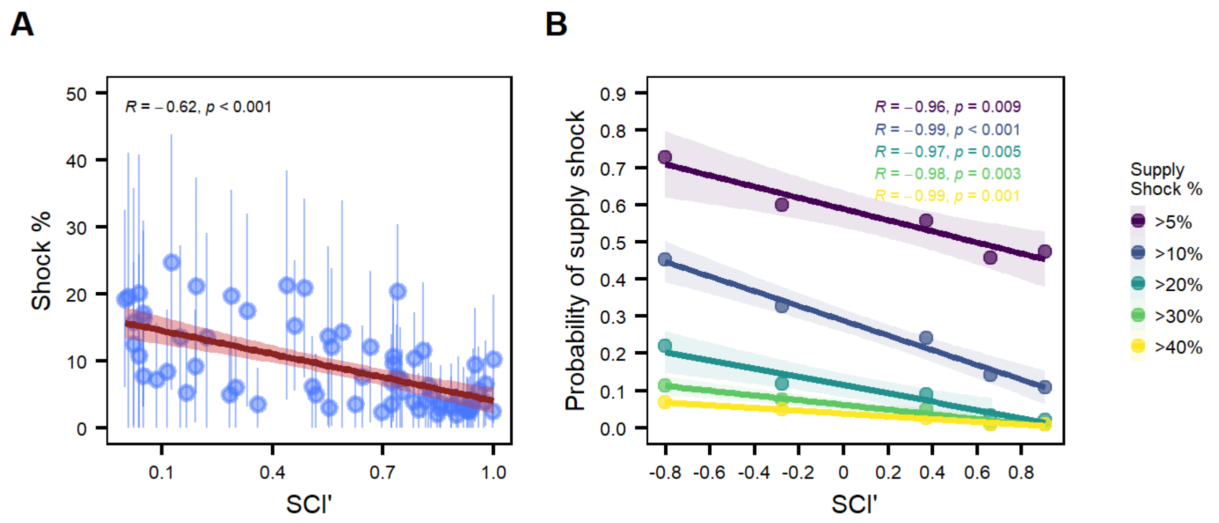

Supplementary Figure 5.

(A) Relationship between the cities' average shock intensity and SCI′ values. For each city, the average shock intensity was calculated from products with $LQ \geq 1$. The vertical lines indicate 1 s.d. of the shock values. (B) Relationship between the cities' probability of supply chain shock and average SCI′ values for five different shock intensity thresholds. For each SCI′ bin, the probability values are obtained by counting the number of cities above a given threshold value and dividing by the total number of cities. The points in the graph show the average SCI′ values within each bin. SCI, vertical supply chain complexity index; SCI′, horizontal supply chain complexity index.

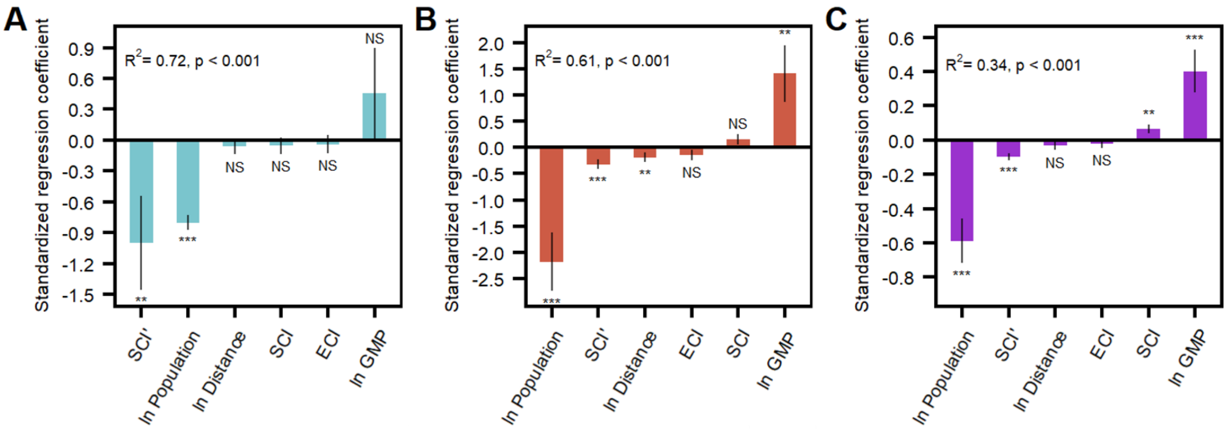

Supplementary Figure 6.

Standardized coefficients associated with the regression of the supply chain shock intensity on the supply chain complexity indices and several control variables [ln population, ln average distance, standardized economic complexity index (ECI), and ln gross metropolitan product (GMP)]. (**A**) Standard coefficients obtained using the average shock intensity of a city's main inflow products as the response variable (n=69). (**B**) Standardize coefficients obtained using the average shock intensity of all inflow products to a city as the response variable (n=69). (**C**) Standardized coefficients obtained using the shock intensity associated with each product-city pair as the response variable (n=2,512). ***, P < 1%; **, P < 5%; and NS (nonsignificant), P > 10%. The standard error is indicated with the black vertical line. SCI, vertical supply chain complexity index; SCI′, horizontal supply chain complexity index.

Supplementary Table 1.

Summary of results for the linear regression fits between the supply chain complexity indices – SCI or SCI′ – and the local city and supply network characteristics.

Variable	Slope		Intercept		R^2	
	**SCI**	**SCI′**	**SCI**	**SCI′**	**SCI**	**SCI′**
ln(Population)	0.80^{***}	-0.43^{**}	14.40^{***}	14.56^{***}	0.20	0.09
ln(GMP)	0.76^{***}	-0.47^{**}	18.33^{***}	18.50^{***}	0.15	0.09
ln(Population density)	0.63^{***}	-0.43^{***}	4.73^{***}	4.88^{***}	0.21	0.15
ECI	0.70^{***}	0.08	0.42^{***}	0.48^{***}	0.21	0.004
ln(In-degree)	0.17^{***}	-0.28^{***}	7.95^{***}	8.02^{***}	0.18	0.75
ln(Out-degree)	0.14^{***}	-0.22^{***}	7.96^{***}	8.02^{***}	0.15	0.57
ln(In-strength)	0.17	-0.68^{***}	25.50^{***}	25.65^{***}	0.01	0.31
ln(Out-strength)	0.18	-0.67^{***}	25.47^{***}	25.61^{***}	0.01	0.27

Note: The number of observations (n) is 69.

$^{*}p < 0.1$, $^{**}p < 0.05$, $^{***}p < 0.01$.

GMP, gross metropolitan product; ECI, economic complexity index; SCI, vertical supply chain complexity index; SCI′, horizontal supply chain complexity index.

Supplementary Table 2.

Variance Inflation Factors (VIFs) for all the predictors used in the regression analyses. For the main effect variables – SCI and SCI′, VIF values are lower than 10, indicating that multicollinearity is not an issue for these variables. The VIF values for the control variables of Population and GMP are greater than 10. However, we include both of these control variables in the regression analyses since they represent key and easily accessible characteristics of cities.

Variable	Variance Inflation Factor (VIF)
ln (Population)	49.50
ln (GMP)	46.15
ECI	1.36
ln (Distance)	1.51
SCI	1.69
SCI′	1.33

GMP, gross metropolitan product; ECI, economic complexity index; SCI, vertical supply chain complexity index; SCI′, horizontal supply chain complexity index.

Supplementary Table 3.

Multivariate multiple linear regression analysis results with the average shock intensity of the main inflow products to a city as the response variable (regression analysis I).

**Variable**	**Coefficient estimates (s.e.) for model:**					
	**1**		**2**		**3**	
	$\ln \hat{S}_r^v$	$\ln \hat{S}_r^h$	$\ln \hat{S}_r^v$	$\ln \hat{S}_r^h$	$\ln \hat{S}_r^v$	$\ln \hat{S}_r^h$
Controls						
ln population [b_1]	−0.89*** (0.23)	−0.45** (0.20)	−0.95*** (0.24)	−0.38 (0.33)	−0.88*** (0.23)	−0.46** (0.20)
ln GMP [b_2]	0.66*** (0.21)	0.18 (0.18)	0.74*** (0.22)	0.22 (0.30)	0.66*** (0.21)	0.20 (0.18)
ECI [b_3]	−0.03 (0.05)	−0.03 (0.04)	−0.07 (0.04)	−0.08 (0.06)	−0.03 (0.05)	−0.03 (0.04)
ln distance [b_4]	−0.12 (0.11)	−0.09 (0.10)	−0.12 (0.12)	−0.10 (0.17)	−0.12 (0.11)	−0.09 (0.10)
Main effects						
SCI [b_5]	−0.11 (0.08)	−0.03 (0.07)			−0.11 (0.08)	−0.05 (0.07)
SCI′ [b_6]	−0.17*** (0.05)	−0.51*** (0.05)			−0.17*** (0.06)	−0.53*** (0.05)
Interaction effect						
SCI × SCI′ [b_7]					−0.02 (0.13)	0.08 (0.11)
Constant [b_0]	3.43*** (1.04)	5.86*** (0.91)	3.01*** (1.10)	4.27*** (1.54)	3.43*** (1.05)	5.84*** (0.91)
R^2	0.473	0.724	0.368	0.147	0.474	0.726
Adjusted R^2	0.423	0.697	0.329	0.094	0.413	0.695
Residual standard error	0.259	0.226	0.279	0.390	0.261	0.227
F statistic	9.39*** (df = 6; 62)	27.09*** (df = 6; 62)	9.32*** (df = 4; 64)	2.77** (df = 4; 64)	7.85*** (df = 7; 61)	23.13*** (df = 7; 61)

Note: The number of observations (*n*) is 69.

*$p < 0.1$, **$p < 0.05$, ***$p < 0.01$.

GMP, gross metropolitan product; ECI, economic complexity index; SCI, vertical supply chain complexity index; SCI′, horizontal supply chain complexity index.

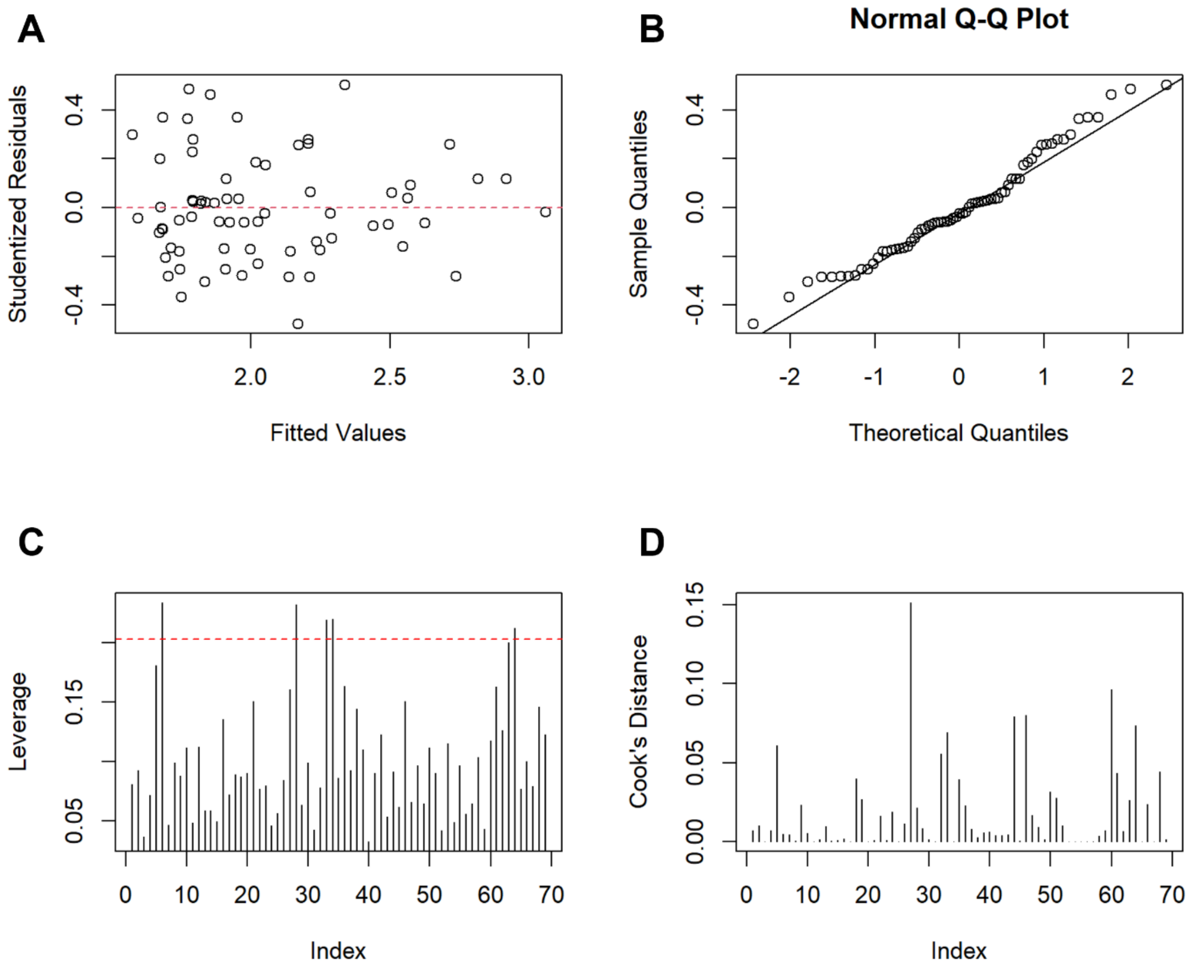

Supplementary Figure 7.

Model diagnostics for regression analysis I with $\ln(S_r^h)$ as the response variable. (A) Plot of studentized residuals versus fitted responses to check the homoscedasticity assumption. (B) Normal quantile-quantile (Q-Q) plot to check the normality assumption. (C) Leverage points for all observations to check for potential outliers. (D) Cook's distances for all observations to check the influence of high-leverage points. All checks are deemed satisfactory.

Supplementary Table 4.

Multiple linear regression analysis results with the average shock intensity of all inflow products to a city as the response variable (regression analysis II).

Variable	Coefficient estimates (s.e.) for model:		
	**1**	**2**	**3**
Controls			
ln population [b_1]	−0.60*** (0.15)	−0.60*** (0.16)	−1.78*** (0.43)
ln GMP [b_2]	0.36** (0.14)	0.38*** (0.14)	−2.06*** (0.77)
ECI [b_3]	−0.06** (0.03)	−0.04 (0.03)	0.07** (0.03)
ln distance [b_4]	−0.12 (0.08)	−0.23*** (0.08)	−0.35 (0.88)
Main effects			
SCI [b_5]	0.08 (0.05)		1.49* (0.83)
SCI′ [b_6]	−0.13*** (0.04)		−1.39*** (0.49)
Interaction effect			
SCI × SCI′ [b_7]			−0.08 (0.96)
Constant [b_0]	5.23*** (0.70)	5.47*** (0.76)	35.55*** (12.33)
R^2	0.611	0.509	0.509
Adjusted R^2	0.573	0.478	0.444
Residual standard error	0.166	0.184	1.96
F statistic	16.24*** (df = 6; 62)	16.57*** (df = 4; 64)	7.77*** (df = 8; 60)

Note: The number of observations (n) is 69.
*$p < 0.1$, **$p < 0.05$, ***$p < 0.01$.

GMP, gross metropolitan product; ECI, economic complexity index; SCI, vertical supply chain complexity index; SCI′, horizontal supply chain complexity index.

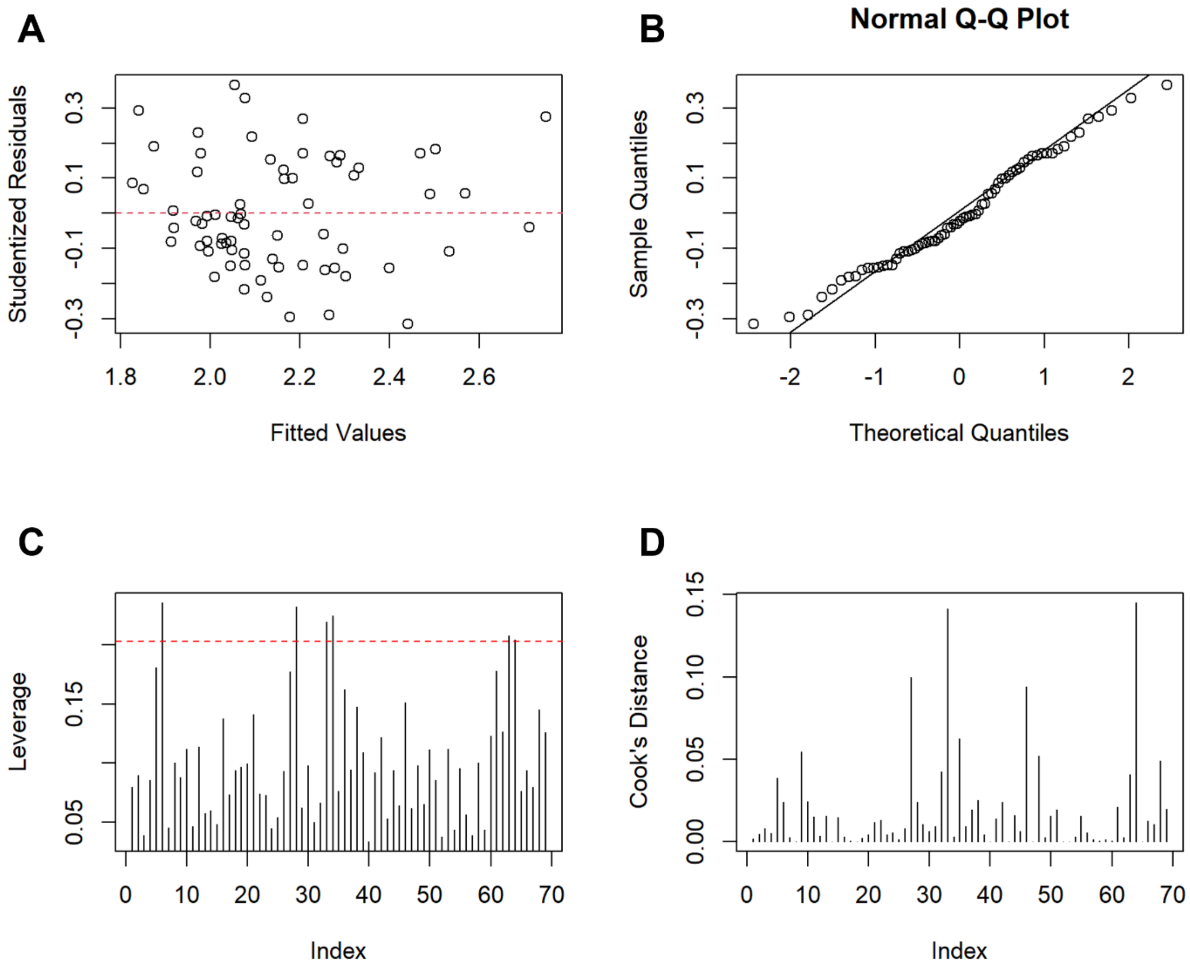

Supplementary Figure 8.

Model diagnostics for regression analysis II with $\ln(S_r)$ as the response variable. (A) Plot of studentized residuals versus fitted responses to check the homoscedasticity assumption. (B) Normal quantile-quantile (Q-Q) plot to check the normality assumption. (C) Leverage points for all observations to check for potential outliers. (D) Cook's distances for all observations to check the influence of high-leverage points. All checks are deemed satisfactory.

Supplementary Table 5.

Multiple linear regression analysis results with the shock intensity from each region-product pair as the response variable (regression analysis III).

Variable	Coefficient estimates (s.e.) for model:		
	1	2	3
Controls			
ln population [b_1]	−0.56*** (0.12)	−0.49*** (0.12)	−0.56*** (0.12)
ln GMP [b_2]	0.35*** (0.11)	0.33*** (0.10)	0.35*** (0.11)
ECI [b_3]	−0.02 (0.02)	−0.005 (0.02)	−0.02 (0.02)
ln distance [b_4]	−0.07 (0.05)	−0.14*** (0.05)	−0.07 (0.05)
Product dummies [$b_{5,1-36}$]	−0.80–1.14*** (0.14–0.19)	−0.80–1.15*** (0.14–0.19)	−0.80–1.14*** (0.14–0.19)
Main effects			
SCI [b_6]	0.11** (0.03)		0.10** (0.05)
SCI′ [b_7]	−0.13*** (0.04)		−0.14*** (0.03)
Interaction effect			
SCI × SCI′ [b_8]			0.03 (0.07)
Constant [b_0]	3.85*** (0.54)	3.76*** (0.54)	3.85*** (0.54)
R^2	0.339	0.329	0.340
Adjusted R^2	0.324	0.314	0.324
Residual standard error	0.718	0.724	0.718
F statistic	22.52*** (df = 42; 1,841)	22.55*** (df = 40; 1,843)	22.00*** (df = 43; 1,840)

Note: The number of observations (*n*) is 2,512.

**p* < 0.1, ***p* < 0.05, ****p* < 0.01.

GMP, gross metropolitan product; ECI, economic complexity index; SCI, vertical supply chain complexity index; SCI′, horizontal supply chain complexity index.

Supplementary Table 6.

Regression coefficients of the 36 product category dummies for the models shown in table S3 (regression analysis III).

Product category	Coefficient estimates (s.e.) for model:					
	1		2		3	
Animal feed	0.22	(0.14)	0.20	(0.14)	0.22	(0.14)
Articles – base metal	-0.25^{*}	(0.14)	-0.26^{*}	(0.14)	-0.25^{*}	(0.14)
Base metals	-0.32^{*}	(0.14)	-0.33^{**}	(0.14)	-0.32^{**}	(0.14)
Basic chemicals	-0.30^{*}	(0.14)	-0.31^{**}	(0.14)	-0.31^{**}	(0.14)
Cereal grains	0.79^{***}	(0.14)	0.81^{***}	(0.14)	0.79^{***}	(0.14)
Chemical products	-0.28^{**}	(0.14)	-0.30^{**}	(0.14)	-0.28^{**}	(0.14)
Coal	0.72^{***}	(0.14)	0.75^{***}	(0.14)	0.72^{***}	(0.14)
Coal – n.e.c.	0.26^{*}	(0.14)	0.25^{*}	(0.14)	0.26^{*}	(0.14)
Crude petroleum	0.86^{***}	(0.19)	0.88^{***}	(0.19)	0.86^{***}	(0.19)
Electronics	−0.17	(0.14)	−0.18	(0.14)	−0.17	(0.14)
Fertilizers	0.39^{***}	(0.14)	0.39^{***}	(0.14)	0.38^{***}	(0.14)
Fuel oils	0.92^{***}	(0.14)	0.94^{***}	(0.14)	0.92^{***}	(0.14)
Furniture	0.13	(0.14)	0.11	(0.14)	0.13	(0.14)
Gasoline	0.66^{***}	(0.14)	0.68^{***}	(0.14)	0.66^{***}	(0.14)
Gravel	0.72^{***}	(0.14)	0.75^{***}	(0.14)	0.72^{***}	(0.14)
Live animals/fish	−0.11	(0.14)	−0.09	(0.14)	−0.11	(0.14)
Logs	1.14^{***}	(0.14)	1.15^{***}	(0.14)	1.14^{***}	(0.14)
Machinery	−0.02	(0.14)	−0.07	(0.14)	−0.02	(0.14)
Meat/seafood	-0.58^{***}	(0.14)	-0.59^{***}	(0.14)	-0.59^{***}	(0.14)
Milled grain products	-0.56^{***}	(0.14)	-0.57^{***}	(0.14)	-0.56^{***}	(0.14)
Motorized vehicles	0.38^{***}	(0.14)	0.36^{***}	(0.14)	0.38^{***}	(0.14)
Natural sands	0.62^{***}	(0.14)	0.65^{***}	(0.14)	0.62^{***}	(0.14)
Newsprint/paper	0.14	(0.14)	0.13	(0.14)	0.14	(0.14)
Nonmetallic mineral products	0.33^{**}	(0.14)	0.32^{**}	(0.14)	0.33^{**}	(0.14)
Nonmetallic minerals	0.82^{***}	(0.14)	0.82^{***}	(0.15)	0.82^{***}	(0.14)
Other agricultural products	−0.01	(0.14)	−0.04	(0.14)	−0.01	(0.14)
Other foodstuffs	-0.30^{**}	(0.14)	-0.31^{**}	(0.14)	-0.30^{**}	(0.14)
Paper articles	-0.80^{***}	(0.15)	-0.80^{***}	(0.15)	-0.80^{***}	(0.15)
Pharmaceuticals	−0.15	(0.14)	−0.16	(0.15)	−0.15	(0.14)
Plastics/rubber	−0.14	(0.14)	−0.16	(0.14)	−0.14	(0.14)
Precision instruments	−0.14	(0.14)	−0.17	(0.14)	−0.14	(0.14)
Printed products	-0.30^{**}	(0.14)	-0.32^{**}	(0.14)	-0.30^{**}	(0.14)
Textiles/leather	-0.67^{***}	(0.14)	-0.69^{***}	(0.14)	-0.67^{***}	(0.14)
Tobacco products	0.68^{***}	(0.14)	0.69^{***}	(0.14)	0.68^{***}	(0.14)
Transport equipment	0.79^{***}	(0.14)	0.78^{***}	(0.14)	0.79^{***}	(0.14)
Wood products	0.42^{***}	(0.14)	0.40^{***}	(0.15)	0.41^{***}	(0.14)

Note: "Alcoholic beverages" is used as the baseline industry.
$^{*}p < 0.1$, $^{**}p < 0.05$, $^{***}p < 0.01$.
n.e.c., not elsewhere classified.

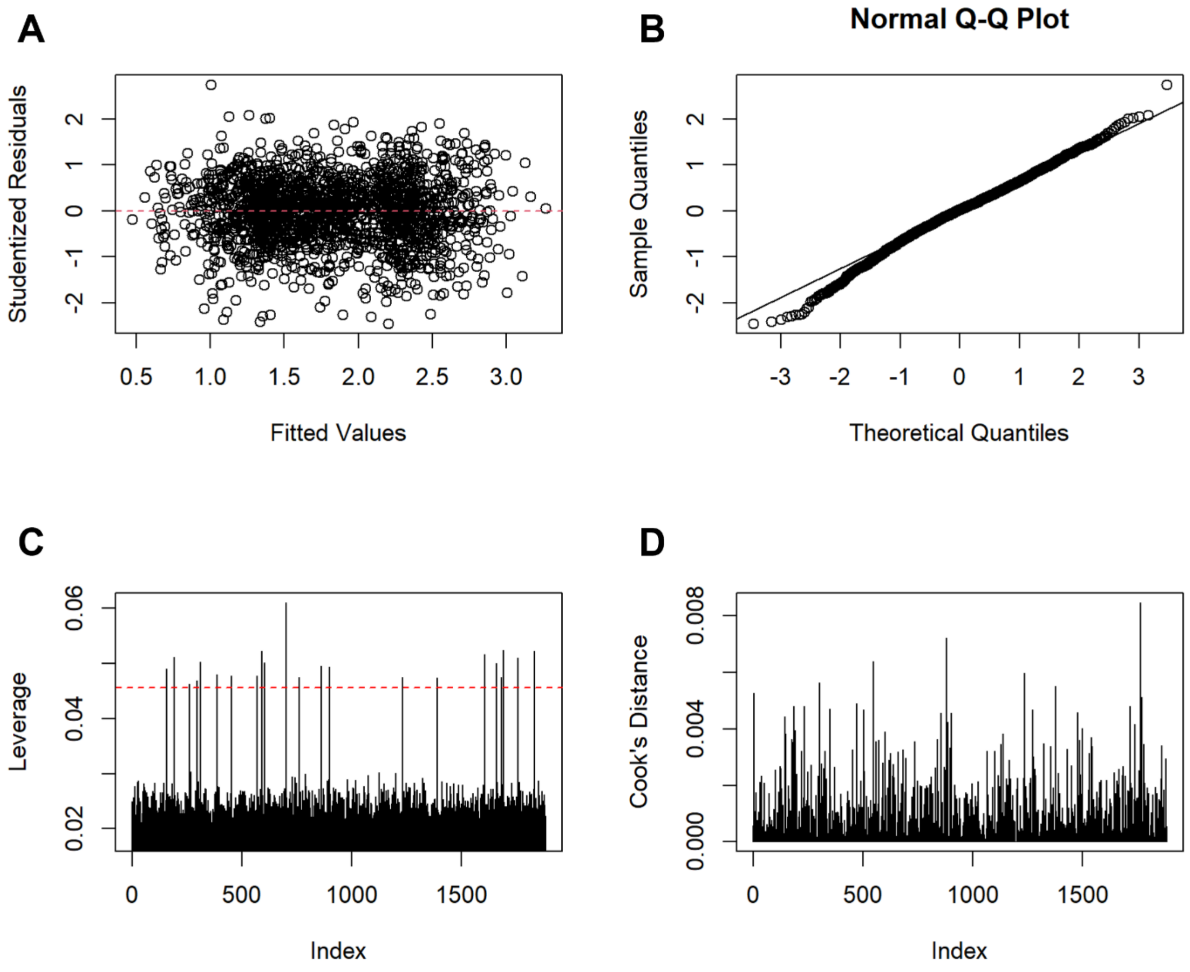

Supplementary Figure 9.

Model diagnostics for regression analysis III with $\ln(S_{rp})$ as the response variable. (A) Plot of studentized residuals versus fitted responses to check the homoscedasticity assumption. (B) Normal quantile-quantile (Q-Q) plot to check the normality assumption. (C) Leverage points for all observations to check for potential outliers. (D) Cook's distances for all observations to check the influence of high-leverage points.

Supplementary Table 7.

Multiple linear regression analysis results with the shock intensity from each region-product pair as the response variable (regression analysis IV.a).

**Variable**	**Coefficient estimates (s.e.) for model:**		
	**1**	**2**	**3**
Controls			
ln population [b_1]	−0.49*** (0.12)	−0.42*** (0.12)	−0.49*** (0.12)
ln GMP [b_2]	0.29*** (0.11)	0.28*** (0.11)	0.30*** (0.11)
ECI [b_3]	−0.02 (0.02)	−0.01 (0.02)	−0.02 (0.02)
ln distance [b_4]	−0.04 (0.05)	−0.11** (0.05)	−0.04 (0.05)
Foreign-sourced supplies (%) [b_5]	0.01*** (0.002)	0.01*** (0.002)	0.01*** (0.002)
Urban-sourced supplies (%) [b_6]	0.005*** (0.001)	0.005*** (0.001)	0.005*** (0.001)
ln production [b_7]	−0.03* (0.01)	−0.03** (0.01)	−0.03* (0.01)
Product dummies [$b_{8,1-36}$]	−0.73–1.09*** (0.14–0.21)	−0.74–1.08*** (0.14–0.21)	−0.73–1.09*** (0.14–0.21)
Main effects			
SCI [b_9]	0.10** (0.04)		0.09** (0.04)
SCI′ [b_{10}]	−0.13*** (0.03)		−0.13*** (0.03)
Interaction effect			
SCI × SCI′ [b_{11}]			0.03 (0.07)
Constant [b_0]	3.35*** (0.57)	3.23*** (0.57)	3.35*** (0.57)
R^2	0.350	0.340	0.350
Adjusted R^2	0.334	0.324	0.334
Residual standard error	0.713	0.718	0.713
F statistic	21.98*** (df = 45; 1,838)	22.06*** (df = 43; 1,840)	21.5*** (df = 46; 1,837)

Note: The number of observations (*n*) is 2,512.
*$p < 0.1$, **$p < 0.05$, ***$p < 0.01$.

GMP, gross metropolitan product; ECI, economic complexity index; SCI, vertical supply chain complexity index; SCI′, horizontal supply chain complexity index.

Supplementary Table 8.

Regression coefficients of the 36 product category dummies for the models shown in table S5 (regression analysis IV.a).

Product category	Coefficient estimates (s.e.) for model:					
	1		2		3	
Animal feed	0.32**	(0.14)	0.30**	(0.15)	0.32**	(0.14)
Articles – base metal	−0.24*	(0.14)	−0.25*	(0.14)	−0.24*	(0.14)
Base metals	−0.28**	(0.14)	−0.29**	(0.14)	−0.29**	(0.14)
Basic chemicals	−0.32**	(0.14)	−0.33**	(0.14)	−0.32**	(0.14)
Cereal grains	0.94***	(0.15)	0.95***	(0.15)	0.94***	(0.15)
Chemical products	−0.26*	(0.14)	−0.27*	(0.14)	−0.26*	(0.14)
Coal	0.88***	(0.17)	0.90***	(0.17)	0.88***	(0.17)
Coal – n.e.c.	0.37***	(0.14)	0.38***	(0.14)	0.37***	(0.14)
Crude petroleum	0.76***	(0.21)	0.75***	(0.21)	0.76***	(0.21)
Electronics	−0.17	(0.14)	−0.18	(0.14)	−0.17	(0.14)
Fertilizers	0.35**	(0.14)	0.33**	(0.14)	0.34**	(0.14)
Fuel oils	0.92***	(0.14)	0.94***	(0.14)	0.92***	(0.14)
Furniture	0.13	(0.14)	0.11	(0.14)	0.13	(0.14)
Gasoline	0.67***	(0.14)	0.70***	(0.14)	0.67***	(0.14)
Gravel	0.63***	(0.15)	0.65***	(0.15)	0.63***	(0.15)
Live animals/fish	−0.02	(0.14)	−0.01	(0.14)	−0.02	(0.14)
Logs	1.09***	(0.15)	1.08***	(0.15)	1.09***	(0.15)
Machinery	0.04	(0.14)	0.01	(0.14)	0.04	(0.14)
Meat/seafood	−0.46***	(0.14)	−0.47***	(0.14)	−0.46***	(0.14)
Milled grain products	−0.52***	(0.14)	−0.54***	(0.14)	−0.53***	(0.14)
Motorized vehicles	0.41***	(0.14)	0.40***	(0.14)	0.41***	(0.14)
Natural sands	0.59***	(0.15)	0.60***	(0.15)	0.59***	(0.15)
Newsprint/paper	0.22	(0.14)	0.22	(0.14)	0.22	(0.14)
Nonmetallic mineral products	0.34**	(0.14)	0.33**	(0.14)	0.34**	(0.14)
Nonmetallic minerals	0.79***	(0.15)	0.77***	(0.15)	0.79***	(0.15)
Other agricultural products	0.06	(0.14)	0.03	(0.14)	0.06	(0.14)
Other foodstuffs	−0.21	(0.14)	−0.21	(0.14)	−0.21	(0.14)
Paper articles	−0.73***	(0.15)	−0.74***	(0.15)	−0.73***	(0.15)
Pharmaceuticals	−0.13	(0.14)	−0.14	(0.14)	−0.13	(0.14)
Plastics/rubber	−0.10	(0.14)	−0.11	(0.14)	−0.10	(0.14)
Precision instruments	−0.14	(0.14)	−0.17	(0.14)	−0.14	(0.14)
Printed products	−0.27*	(0.14)	−0.29**	(0.14)	−0.27*	(0.14)
Textiles/leather	−0.68***	(0.14)	−0.71***	(0.15)	−0.68***	(0.14)
Tobacco products	0.61***	(0.14)	0.61***	(0.14)	0.61***	(0.14)
Transport equipment	0.78***	(0.14)	0.76***	(0.14)	0.78***	(0.14)
Wood products	0.47***	(0.14)	0.46***	(0.14)	0.47***	(0.14)

Note: “Alcoholic beverages” is used as the baseline industry.
*$p < 0.1$, **$p < 0.05$, ***$p < 0.01$.
n.e.c., not elsewhere classified.

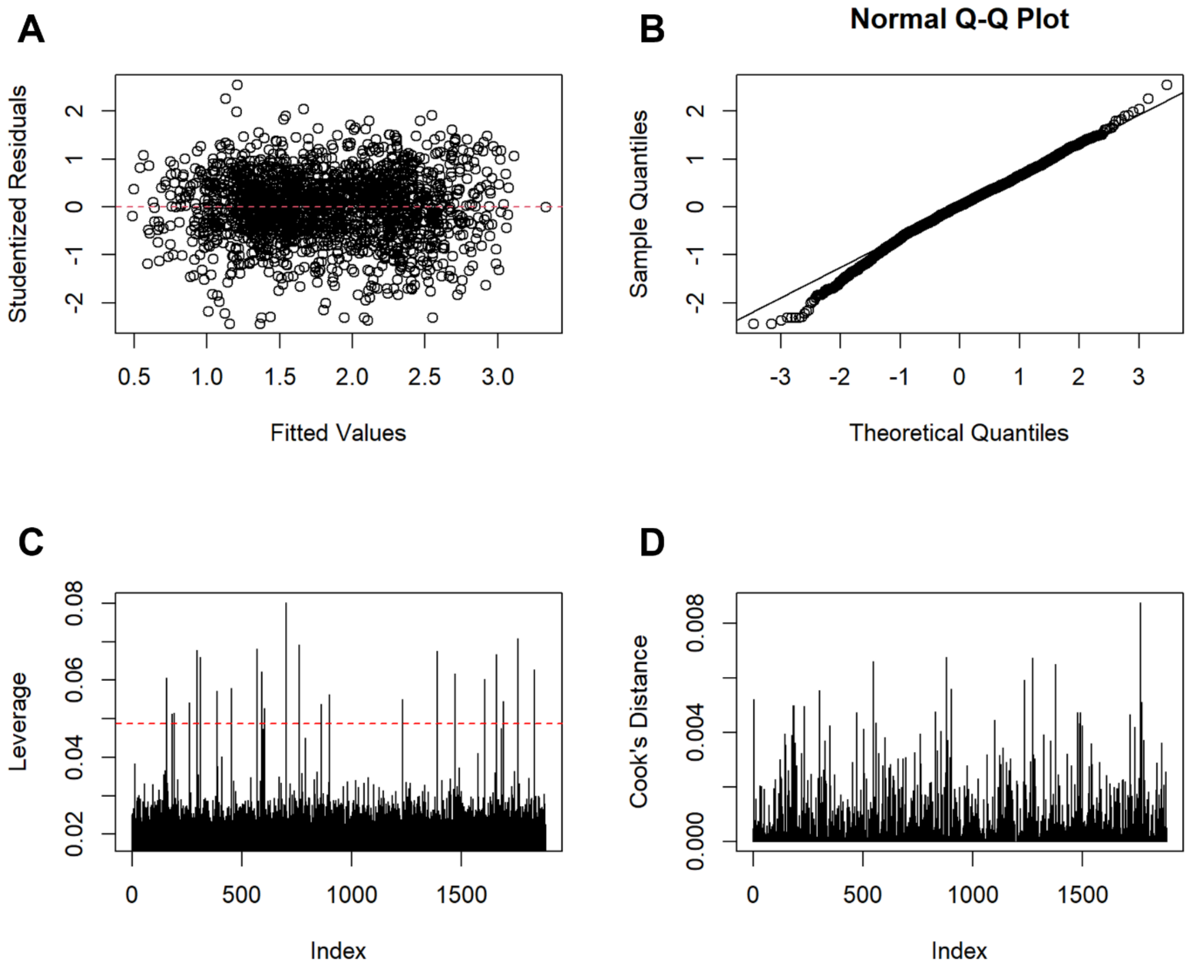

Supplementary Figure 10.

Model diagnostics for regression analysis IV.a with $\ln(S_{rp})$ as the response variable. (A) Plot of studentized residuals versus fitted responses to check the homoscedasticity assumption. (B) Normal quantile-quantile (Q-Q) plot to check the normality assumption. (C) Leverage points for all observations to check for potential outliers. (D) Cook's distances for all observations to check the influence of high-leverage points.

Supplementary Table 9.

Multiple linear regression analysis results with the shock intensity from each region-product pair as the response variable (regression analysis IV.b).

**Variable**	**Coefficient estimate (s.e.)**
Controls	
ln population [b_1]	−0.57*** (0.13)
ln GMP [b_2]	0.36*** (0.11)
ECI [b_3]	−0.06*** (0.02)
ln distance [b_4]	−0.02 (0.05)
Foreign-sourced supplies (%) [b_5]	0.006*** (0.002)
Urban-sourced supplies (%) [b_6]	0.005*** (0.001)
ln production [b_7]	−0.03* (0.01)
Product dummies [$b_{8,1-36}$]	−0.73–1.09*** (0.14–0.21)
Main effects	
SCI [b_9]	0.14*** (0.04)
SCI′ [b_{10}]	−0.13*** (0.03)
Constant [b_0]	3.21*** (0.58)
R^2	0.361
Adjusted R^2	0.345
Residual standard error	0.705
F statistic	23.13*** (df = 45; 1,838)

Note: The number of observations (*n*) is 2,512.
*$p < 0.1$, **$p < 0.05$, ***$p < 0.01$.
GMP, gross metropolitan product; ECI, economic complexity index; SCI, vertical supply chain complexity index; SCI′, horizontal supply chain complexity index.

Supplementary Table 10.

Regression coefficients of the 36 product category dummies for the model shown in table S7 (regression analysis IV.b).

**Product category**	**Coefficient estimate**	**s.e.**
Animal feed	0.09	0.14
Articles – base metal	-0.37^{***}	0.14
Base metals	-0.32^{**}	0.14
Basic chemicals	-0.36^{***}	0.14
Cereal grains	0.87^{***}	0.15
Chemical products	-0.38^{***}	0.14
Coal	0.78^{***}	0.17
Coal – n.e.c.	0.31^{**}	0.14
Crude petroleum	0.55^{***}	0.21
Electronics	−0.18	0.14
Fertilizers	0.42^{***}	0.14
Fuel oils	0.84^{***}	0.14
Furniture	0.13	0.14
Gasoline	0.57^{***}	0.14
Gravel	0.58^{***}	0.14
Live animals/fish	−0.06	0.14
Logs	1.08^{***}	0.16
Machinery	−0.07	0.14
Meat/seafood	-0.49^{***}	0.14
Milled grain products	-0.66^{***}	0.14
Motorized vehicles	0.27^{*}	0.14
Natural sands	0.50^{***}	0.15
Newsprint/paper	0.14	0.14
Nonmetallic mineral products	0.16	0.14
Nonmetallic minerals	0.72^{***}	0.14
Other agricultural products	−0.04	0.14
Other foodstuffs	-0.34^{**}	0.14
Paper articles	-0.92^{***}	0.14
Pharmaceuticals	-0.23^{*}	0.14
Plastics/rubber	−0.20	0.14
Precision instruments	−0.12	0.14
Printed products	-0.37^{***}	0.14
Textiles/leather	-0.61^{***}	0.14
Tobacco products	0.51^{***}	0.14
Transport equipment	0.62^{***}	0.14
Wood products	0.30^{**}	0.14

Note: “Alcoholic beverages” is used as the baseline industry.
$^{*}p < 0.1$, $^{**}p < 0.05$, $^{***}p < 0.01$.
n.e.c., not elsewhere classified.

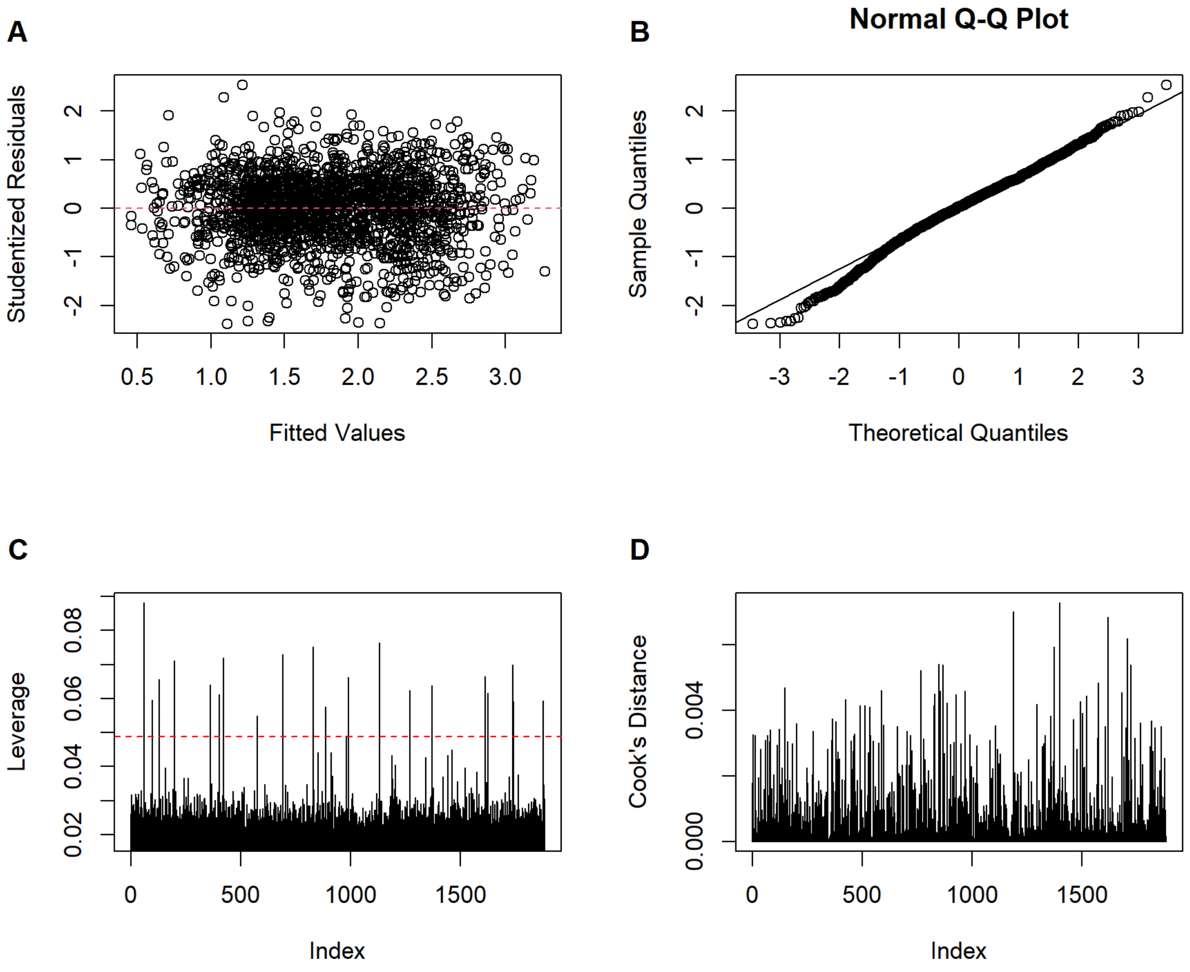

Supplementary Figure 11.

Model diagnostics for regression analysis IV.b with $\ln(S_{rp})$ as the response variable. (A) Plot of studentized residuals versus fitted responses to check the homoscedasticity assumption. (B) Normal quantile-quantile (Q-Q) plot to check the normality assumption. (C) Leverage points for all observations to check for potential outliers. (D) Cook's distances for all observations to check the influence of high-leverage points.
